# Gut-initiated alpha synuclein fibrils drive parkinsonism phenotypes: temporal mapping of REM sleep behavior disorder-like and other non-motor symptoms

**DOI:** 10.1186/s40035-026-00536-6

**Published:** 2026-03-10

**Authors:** Daniel Dautan, Wojciech Paslawski, Sergio G. Montejo, Daniel C. Doyon, Valentina I. Brioschi, Roberta Marongiu, Michael G. Kaplitt, Rong Chen, Valina L. Dawson, Xiaoqun Zhang, Ted M. Dawson, Per Svenningsson

**Affiliations:** 1 Department of Clinical Neuroscience, Karolinska Institute, Stockholm, Sweden; 2 Aligning Science Across Parkinson’s (ASAP) Collaborative Research Network, Chevy Chase, MD 20815 USA; 3 Department of Neurological Surgery, Weill Cornell Medicine, New York, NY USA; 4 Neuroregeneration and Stem Cell Programs, Institute for Cell Engineering, The Johns Hopkins University School of Medicine, Baltimore, MD USA; 5 Department of Neurology, The Johns Hopkins University School of Medicine, Baltimore, MD USA; 6 Department of Physiology, Johns Hopkins University School of Medicine, Baltimore, MD 21205 USA; 7 Solomon H. Snyder Department of Neuroscience, The Johns Hopkins University School of Medicine, Baltimore, MD USA; 8 Department of Pharmacology and Molecular Sciences, The Johns Hopkins University School of Medicine, Baltimore, MD USA

**Keywords:** Parkinson’s disease, Alpha-synuclein, Gut-brain axis, REM sleep behavior disorder, Dopamine

## Abstract

**Background:**

Parkinson’s disease (PD) is a progressive neurodegenerative disorder marked by both motor and non-motor symptoms. Although non-motor features such as gastrointestinal and sleep disturbances often precede motor impairments and are critical to PD pathogenesis, the mechanisms underlying their onset and progression remain insufficiently characterized.

**Methods:**

To investigate the sequential development of motor and non-motor symptoms in a model of experimental parkinsonism, we injected alpha-synuclein (αSyn) preformed fibrils (PFFs) into the duodenum and antrum of wild-type mice, establishing a gut-brain axis model of PD. We performed whole-brain anatomical mapping of αSyn-PFF propagation and assessed behavioral alterations at multiple time points post-injection. Correlations between anatomical spread and behavioral changes, particularly sleep, were further validated through *SNCA* overexpression or local αSyn-PFF injections in the substantia nigra, combined with dual-wavelength fiber photometry, behavioral assays, and histological analyses.

**Results:**

Injection of αSyn-PFFs into the gastrointestinal tract of wild-type mice led to a progressive spread of pathological αSyn throughout the central nervous system, in temporal association with distinct motor and non-motor phenotypes. These findings provide translational validity of the gut-brain model, mirroring the clinical progression seen in many PD patients. In two established αSyn-based PD models, dual-wavelength fiber photometry that monitors dopamine and acetylcholine release in the striatum, demonstrated a central role for dopamine dysfunction in modulating sleep architecture, particularly in relation to REM sleep without atonia, consistent with REM sleep behavior disorder (RBD)-like manifestations in PD.

**Conclusion:**

This work provides a detailed characterization of the progressive and multisystem nature of experimental parkinsonism, highlighting the interplay between αSyn pathology, gut-brain signaling, and the onset of non-motor disturbances, with a particular focus on RBD-like alterations in sleep.

**Supplementary Information:**

The online version contains supplementary material available at 10.1186/s40035-026-00536-6.

## Background

Parkinson’s disease (PD) is diagnosed based on the presence of bradykinesia, rigidity, and tremor. Non-motor symptoms in PD encompass a diverse array of manifestations that can occur years before motor symptoms and impact patients’ lives [1, 2]. Rapid eye movement (REM) sleep behavior disorder (RBD) is a prodromal manifestation with the highest likelihood to phenoconvert to PD [3].

The pathological hallmark of PD is the accumulation of misfolded and aggregated alpha-synuclein (αSyn) that is critical in Lewy body formation [4, 5]. Aggregated forms of αSyn have increased phosphorylation at the serine 129 (pS129) residue [6], although pS129 phosphorylation may also play a physiological role in synaptic function [6]. Some evidence suggests that αSyn spreads from the gut throughout the vagus nerve to the central nervous system (CNS) [7, 8]. This spreading of pathologic αSyn along the gut-brain axis may modulate the progressive emergence of non-motor symptoms in PD [9].

Most research on experimental Parkinsonism models focuses on the later stages of PD (i.e., motor symptoms) and neglects premotor stages. The injection of αSyn preformed fibrils (PFFs) in the stomach/duodenum represents a clinically relevant model offering insights into the progression from early (i.e. non-motor symptoms) to later stages of PD [10, 11].

In the present study, we conducted comprehensive behavioral and physiological assessments of non-motor and motor symptoms in mice injected with αSyn-PFFs in the stomach/duodenum at different time points. The results demonstrated a progressive accumulation of pS129-αSyn correlating with the appearance of non-motor symptoms, particularly impaired sleep architecture and RBD-like signs along with dopamine dysfunction. In subsequent experiments, we therefore targeted the nigrostriatal pathway with αSyn-PFFs or AAV-αSyn and confirmed a role for the substantia nigra pars compacta (SNc) in mediating RBD-like signs.

## Materials and methods

### Data sharing

Detailed protocols are available on the https://www.protocols.io platform at the links provided in the Materials and methods section. Prism files, which include all individual values for all tests, detailed statistical analyses, raw and processed images, photometry, sleep recordings, all MATLAB and ImageJ scripts, and behavioral videos, are available at 10.5281/zenodo.10822457. Raw confocal images with file sizes too large to deposit to Zenodo are available upon request to the corresponding authors or kaplittlab@med.cornell.edu. The data, code, protocols, and key lab materials used and generated in this study are listed in a Key Resources Table alongside their persistent identifiers at 10.5281/zenodo.10822457.

### Chemicals and antibodies

Unless stated otherwise, all chemicals were purchased from Sigma Aldrich (Merck KGaA, Germany) and were of analytical grade. All solutions were prepared using Milli Q deionized water (Millipore, MA).

### Animals

Wild-type C57BL/6 mice (bred in-house from Jackson Laboratory, Sulzfeld, Germany, cat #000664; RRID: ISMR_JAX:000664) were maintained in the animal facility at the Karolinska Institute, Sweden, adhering to the guidelines set forth by the local ethical committee at Stockholm Norra Djurförsöksetiska Nämnd (3218-2022, 24297-2022) and the European Communities Council Directive (86/609/EEC). The mice were housed in rooms with a 12-h light cycle (lights on at 07:00) and controlled temperature (20 °C) and humidity (53%). Throughout the entire experimental period, animals had unrestricted access to food and water. To minimize within-experiment variability, experimental animals and their respective controls were sourced from the same litter, ensuring identical age and housing conditions. Notably, both male and female mice were handled consistently across all experiments.

Animals were distributed as follows: 7 animals (4 females, 3 males) received a single injection (2 needle penetrations for 4 different sites of infusion) of monomeric αSyn 2 weeks before behavioral testing (4 more males were added for sleep recording); 9 animals (6 females, 3 males) received a single injection of monomeric αSyn which was performed 7 months prior to behavioral testing; 6 animals (3 females, 3 males) received a single injection of αSyn-PFF 2 weeks before behavioral testing; 8 animals (4 females, 4 males) received a single injection of monomeric αSyn one month before behavioral testing; 10 animals (5 females, 5 males) received a single injection of αSyn-PFF 3 months before behavioral testing; 21 animals (8 females, 13 males) received a single injection of αSyn-PFF 6 months before behavioral testing; 10 animals (6 females, 4 males) received a single injection of αSyn-PFF 7 months before behavioral testing.

### αSyn purification and αSyn-PFF preparation

αSyn plasmid (full-length pRK172 vector) was transformed into *E. coli* bacteria genetically modified to prevent LPS-mediated immune response. The starter culture was maintained at − 80 °C until use to generate αSyn monomer. When needed, the starter culture was transferred to LB medium with ampicillin overnight at 37 °C before being resuspended in high-salt buffer and EDTA solution. Next, the bacterial cells were broken using a high-pressure homogenizer and boiled for 15 min to precipitate other proteins. Following cooling on ice and spun at 6000× *g* for 20 min, the supernatant was dialyzed and filtered through Amicon Ultra centrifuge filter (100 kDa). Each fraction was then checked using SDS-PAGE and Coomassie Blue staining to collect αSyn bands (~ 15 kDa). Next, the fractions were concentrated and stored at − 80 °C until use for generating fibrils.

When required for fibril generation, the aliquot was centrifuged at 12,000× *g* at 4 °C, the supernatant was removed, and protein concentration was determined using a BCA assay. Monomeric protein was then adjusted to 5 mg/mL and shaken for 7 days at 37 °C with 1000 rpm. Following that, fibrils were validated with Thioflavin T assay/Sedimentation assay before being kept at − 80 °C until use for injections.

When required for injection, αSyn-PFF was diluted to 2 mg/mL by adding sterile PBS and sonicated at 20% amplitude, for a total of 60 pulses (0.5 s on/off cycle) with a short break every 10 pulses. For all αSyn-PFF preparations, a small aliquot was processed for transmission electron microscopy to validate αSyn-PFF quality, while the remaining was used for injection. During injection, sonicated αSyn-PFF aliquots were kept for a maximum of 6 h to avoid re-aggregation. To avoid within-subject variability, all experiments were performed at the same time using the same lot of monomeric αSyn and αSyn-PFF. See 10.17504/protocols.io.dm6gp3nm5vzp/v1.

### Intra-muscularis layer injection in the stomach and proximal duodenum

At 2 months of age, mice were anesthetized with 2% isoflurane (in O_2_) and positioned supine with extended limbs. After shaving the abdominal area, a transverse incision was made on the right side, just below the thoracic cage. Subsequently, a second smaller incision, but similar to the first, was made through the abdominal muscles after dissociation from the skin.

Using small, blunt forceps, the liver was gently elevated, exposing the stomach, which was carefully drawn out of the abdominal cavity. After identifying the curvature of the stomach and the pyloric sphincter, a 10 µL Hamilton syringe loaded with 10 µL of αSyn-PFF at a concentration of 2.5 µg/µL was inserted along the pyloric canal. In a single penetration, two discrete injections (2.5 µL each) were administered along the lesser curvature of the stomach within the muscularis layer.

A second penetration, perpendicular to the antrum, targeted the muscularis layers of the duodenum adjacent to the sphincter, where two injections (2.5 µL each) were delivered while slowly withdrawing the needle. Following injection, the syringe was withdrawn gradually to prevent any backflow. Abdominal muscles were sutured using a lock-stitch technique to minimize surgical complications. The skin was closed with simple interrupted stitches and surgical glue (Vetbond, 3M, Saint Paul, MN). Post-surgery, animals were transferred to clean cages with temperature monitoring for recovery. Over the subsequent 3 days, all animals received analgesic treatment (ketoprofen). See: 10.17504/protocols.io.rm7vzjm62lx1/v1.

### Stereotaxic surgery to target SNc or striatum

Animals were injected bilaterally with virus and αSyn-PFF for behavioral and electrophysiology experiments, or unilaterally for anatomical mapping. During surgery, adult mice (~ 2–3 months old) were anesthetized with isoflurane (~ 2% in O_2_) and then placed on a stereotaxic apparatus (Kopf Instruments, Tujunga, CA). Following shaving the skull and opening an incision on the scalp, a small drill hole was made above the injected structures. Viral vectors or αSyn-PFF was injected using a Hamilton Syringe (NanoNeurons #7001) connected to a nanoinjector (WPI, Sarasota, FL). Overexpression of hSNCA was achieved with the injection of AAV6-CAG-αSyn, with a titer of 2.3 × 10^11^ viral particles per microliter. All injections were performed over 10 min, followed by 5 min for diffusion. Following injection, the syringe was slowly withdrawn, and the scalp was sutured. Animals were allowed to recover for 2 weeks after surgery.

Stereotaxic injections were aimed at injecting into the SNc (250 nL, AP: −3.6 mm, ML: 1.5 mm, DV: 4.2 mm) and striatum (500 nL, AP: +0.5 mm, ML: ±1 and 2 mm, DV: 2.5 and 3.5 mm). All coordinates were defined from the Bregma and the surface of the brain. For injection into the SNc or striatum, staining for tyrosine hydroxylase (TH) was used to define the appropriate targeting of the structure. See: 10.17504/protocols.io.j8nlk1nz5g5r/v1.

### Behavioral testing

For all behavioral experiments, animal cages were transferred to the behavioral antechamber one hour before testing for acclimatization. Male and female animals were tested separately, with a minimum of 10 min allocated as a buffer time for transitions between sexes. Following each trial, the apparatus underwent thorough cleaning using a 50% ethanol solution to prevent any potential cross-contamination between animals from different cages.

Consistency in testing conditions was maintained by conducting all experiments at the same time of day, with a permissible variation of up to 4 h, either in the morning or afternoon. This approach was adopted to mitigate variability associated with diurnal cycles.

### Olfactory discrimination test

Animals were transferred to a 30 × 30 cm^2^ gray open field chamber equipped with overhead housing lights and a camera recording system positioned above the apparatus. The camera was connected to a behavior-tracking software (EthoVision XT, RRID:SCR_000441) for precise data analysis. To prevent day-to-day habituation, no buffer habituation sessions were implemented.

On the first day of the experiment, mice were introduced to the apparatus for 20 min, accompanied by a 5 cm plexiglass circle cage filled with chocolate-flavored pellets (05684, Bio-Serv, San Diego, CA). On the subsequent day, a different apparatus was utilized, and the chocolate-flavored pellets were replaced with cotton pads infused with 1 mL of a 1% solution of 2,3,5-trimethyl-3-thiazoline (TMT, Sigma Aldrich) in water.

For each experimental session, parameters such as the distance travelled, time spent in the center, average speed, and time spent in the corner associated with the olfactory stimulus were extracted from the tracking software. The olfactory discrimination index was calculated as the ratio of time spent in the corner linked with chocolate flavor to the time spent in the corner associated with TMT. A ratio of 1 was considered indicative of no significant preference for either odor. See: 10.17504/protocols.io.36wgq3wwxlk5/v1.

### Sucrose preference test

Individually housed mice in single cages were provided access to two novel bottles, distinct from those used for husbandry. One bottle contained a 1% sucrose solution, while the other contained regular water. After 24 h, each bottle was weighed, and the weights were compared to the initial measurements to determine water and sucrose consumption. The ratio of water to sugar consumption was then expressed as the sucrose preference index. See: 10.17504/protocols.io.4r3l22wwxl1y/v1.

### Dark–light box

A 45 × 20 × 20 cm^3^ custom box divided into 1/3 dark (covered with a lid, < 5 lx) and 2/3 white (~ 100 lx) was used for the experiment. Animals were released in the dark compartment before positioning the lid and left to explore for 6 min. All experiments were video-recorded, and manual scoring was done for the time in the light box as well as the number of times the mice entered the light box. See: 10.17504/protocols.io.ewov19jz7lr2/v1.

### Rotarod 40 RPM or 20 RPM with docking

The animals were positioned on a Rota-Rod apparatus (Ugo Basile, Germonio, Italy, Cat # 47650, https://ugobasile.com/products/categories/motory-coordination/rotarod-for-mice-and-rats) with the receptacle platform in the upward position. The session was repeated three times on three consecutive days at a consistent time of day (09:00–12:00). Mice were allowed to acclimate for a few seconds before initiating the rotation protocol. Up to five mice were tested simultaneously if they originated from the same cage.

Upon commencing the protocol for regular rotarod, the speed was increased to 40 rotations per minute (40 rpm) and maintained at this speed for 120 s. For each mouse, the time to fall (determined by the receptacle) or the time for the mouse's body to complete three full rotations (head down) around the beams was recorded.

For the rotarod with docking, the speed was increased to 20 rotations per minute (20 rpm) and maintained at this speed for five rotations before stopping. Subsequently, the platform rotated in the opposite direction for five rotations at 20 rpm (docking). For each mouse, the time to fall (determined by the receptacle) or the time for the mouse's body to complete three full rotations (head down) around the beams was noted. See: 10.17504/protocols.io.3byl4qo5zvo5/v2.

### Bedding test

Mice were individually housed for a minimum of 48 h before testing. On the test day at 09:00, the mice were transferred to a clean cage with ad libitum access to food and water, featuring a 5 × 5 cm^2^ cotton pad positioned on the floor. After 24 and 48 h, a picture was taken of the apparatus, and a bedding quality score (on a scale of 0 to 5 points) was assigned based on specific parameters: 0, mouse did not touch the pad; 1, mouse unfolded the pad; 2, mouse folded the pad in a corner; 3, mouse folded the pad in a corner with bite marks; 4, mouse initiated shredding of the pad and created a nest; and 5, the pad was shredded, with a visible proper nest. See: 10.17504/protocols.io.n2bvj3ko5lk5/v1.

### Descending pole test

Mice were positioned on top of a wooden pole (50 cm long, ~ 1 cm diameter) leading to a clean cage. It is noteworthy that the mice were not subjected to training to prevent contamination of the learning experience. The mice were placed on the pole facing upward, and the time taken from head turning downward to forelimb touching the bedding was recorded via video analysis. See: 10.17504/protocols.io.4r3l2qqj3l1y/v1.

### Descending inclined platform test

At the initiation of the test, mice were positioned on the horizontal section of a 60-cm long, 45° inclined platform featuring a 1 cm grid. Subsequently, mice were gently prompted to the starting point of the inclined path and allowed to move down freely. The time taken to reach the clean cage positioned at the bottom was then measured utilizing video-recorded sessions. Importantly, mice were not subjected to training or habituation on the platform to prevent any contamination of learning effects. For all animals, 5 different frames were randomly selected for the period of mouse descending the platform. Each frame was then used to determine the distance between hindlimbs expressed as a number of grid frames. Next, the number of grid frames was converted to centimeters. See: 10.17504/protocols.io.14egn3ko6l5d/v2.

### Operant behavior

Operant behavior testing was conducted in individually housed mice without access to regular food. In each animal cage, a FED3.0 operant box was positioned [12]. To prevent wood bedding from entering the nose poke, the amount of bedding provided to each animal was deliberately reduced. The FED3.0 operant box was defined with two nose pokes using infrared beams to determine nose entry and a food pellet dispenser was positioned in the middle.

Initially, mice were exposed to FED3.0 for 24 h with 6 g of chow pellets placed on the floor. In the following two days, mice were exposed to free-feeding sessions where 20 mg sugar pellets (BioServ, #f07595, https://www.bio-serv.com/product/DPP_Sucrose.html) were randomly delivered to habituate the mice. The next three days involved a switch to a FR1 protocol starting at 09:00. Each correct poke was paired with an auditory cue and the delivery of a single sugar pellet, while an incorrect poke was paired with a visual cue (blue LED positioned below the pellet dispenser). When a pellet was delivered, both correct and incorrect nose pokes became inactive to prevent multiple pellet deliveries.

Following three consecutive sessions of FR1, the protocol was increased to Fixed Ratio 3 (FR3) for one day. In this protocol, when an animal produced three correct nose pokes, a 20 mg pellet was delivered with the same auditory cue. Any incorrect poke was paired with a visual cue to indicate errors. Similar to FR1, when a pellet was delivered, both correct and incorrect nose pokes were made inactive to avoid multiple pellet deliveries.

Finally, on the last day, the protocol was switched to a "Follow the Light" protocol for 24 h. In this protocol, correct and incorrect sides were alternated randomly using an FR1 schedule. The poke associated with pellet delivery was indicated by a yellow LED turning ON in the nose poke. When a poke occurred on the correct side, a 20 mg sugar pellet was delivered with an auditory cue, while an incorrect poke was paired with a visual cue. Similar to the previous protocols, when a pellet was delivered, both correct and incorrect nose pokes became inactive to prevent multiple pellet deliveries. The number of correct and incorrect pokes and pellets collected within 10 s of delivery were then extracted for further analysis. See: dx.doi.org/10.17504/protocols.io.j8nlk8b31l5r/v1.

### Telemetry implantation

Mice underwent deep anesthesia with 2% isoflurane in O_2_ before being secured in a stereotaxic frame (Kopf Instruments). The skin over the skull was incised and retracted to expose the skull. An electroencephalogram (EEG) electrode (MCS1×2; Agnthos, Lidingo, Sweden) was then screwed into the skull above the frontal cortex (AP: +1.5, ML: 1.0), and a reference electrode was secured into the skull above the cerebellum. Electromyogram (EMG) electrodes were positioned in the nuchal muscles on the same side with approximately 1-mm spacing; these electrodes were then affixed using small heat-shrink tubing. Subsequently, a sizable chamber was created in the abdominal area to accommodate the wireless telemetry recording device (F20-EET;DSI, Dallas, TX). The cavity was thoroughly flushed with saline to prevent complications, and the skull electrodes were secured using dental cement (Paladur; Kulzer, Wehrheim, Germany). Finally, the skin was sutured and covered with surgical glue. For all animals, EEG was recorded on channel 2, while EMG was recorded on channel 1 of the wireless telemetry device. See: https://www.protocols.io/view/procedure-for-eeg-surgery-kxygx3dxog8j/v1.

### Telemetry recording

Following a recovery period of at least one week post-surgery, animals were transferred to the sleep-recording room and allowed to acclimate for a minimum of 48 h. During the recordings, animals were group-housed with 1–2 cage mates throughout the entire procedure, enabling the characterization of sleep independently of any social stress factors. On the day of the recording, the telemetry device was activated using a strong magnet, and the cages were placed on the telemetry receiver (DSI, https://www.datasci.com/products/software/neuroscore). A total of 16 animals were recorded simultaneously. EEG, EMG, and movement activity were recorded via telemetry using the Neuroscore v3.0 software (DSI) at a frequency of 500 Hz for EEG/EMG and 1 Hz for activity. Mice were recorded for 24 h without interruption in a soundproof room, with ad libitum access to food and water.

### Sleep data analysis

Sleep data files were imported into the Neuroscore V3.0 software and visually inspected. Data with interruptions, noise, or issues related to battery status were excluded from further analyses. Subsequently, the DSI-provided "sleep scoring 2" script was utilized for analysis, incorporating EEG, EMG, and activity channels for scoring (Tools – Rodent scoring 2 visual Tuning).

For our analyses, specific power levels were considered indicative of different sleep stages:

- Delta power levels with a delta ratio of 0.5 were associated with a maximum probability of REM/Wake, and a non-REM (NREM) delta ratio of 1 represented a maximum probability of NREM.
- Theta power levels with a theta-to-delta ratio of 1.3 indicated a maximum probability of Wake/NREM, and a theta-to-delta ratio of 3 indicated a maximum probability of REM.
- EMG power levels with an EMG ratio of 1.1 were considered representative of a maximum probability of REM to NREM, and an EMG ratio of 2.4 indicated a maximum probability of Wake.
- An activity count of 0.1 was defined for the active wake phase. To avoid separating different wake stages, the scoring rules combined active wake and wake based on the activity channel.

Artifact detection thresholds were set on a per-recording basis, with most recordings having thresholds set to 0.5 mV for both EEG and EMG.

Stage transition probabilities based on EEG, EMG, and activity were defined as follows:

- Wake to NREM: 90%
- Wake to REM: 90%
- NREM to Wake: 80%
- NREM to REM: 90%
- REM to Wake: 70%
- REM to NREM: 80%

Delta oscillation was determined within a power band of 0.5 to 4 Hz, while Theta oscillation was within the 6 to 9 Hz range. The contributing factors of Delta, Theta/Delta, and EMG were considered equally important for all recordings. Sleep scoring was then obtained within 20 s windows, and any recording with more than 1% artifacts was excluded from further analyses. See 10.17504/protocols.io.yxmvm3rrbl3p/v1.

### REM sleep behavior disorder scoring and periodogram analyses

Following the scoring of sleep stages, EMG was converted into the root mean square (RMS) using the signal grid function. For all recordings, a signal grid was generated, including the timestamps (20-s windows) of the Greenwich Mean Time, the RMS of the EMG, the sleep scoring, and the EEG. Additionally, a periodogram Power Band (PB) for each 20-s window was obtained within the 0.3–80 Hz band range. The analysis employed a 10/1024 FFT order, an overlap of 50%, and a Hamming spectral function applied to the window. PB values were expressed as a relative percentage of the power band value. As mentioned above, Delta oscillation was determined within the 0.5–4 Hz window, Theta within the 4–8 Hz window, Alpha within the 8–12 Hz window, Sigma within the 12–16 Hz window, Beta within the 16–24 Hz window, Low Gamma within the 24–49 Hz window, and High Gamma within the 51–80 Hz window.

The signal grid was then exported to Excel for further analysis. In Excel, the RMS-EMG was first converted to the z-score over the entire recording. A COUNTIF function was then used to count the number of REM events (scored as P), awake events (scored as W), or NREM events (scored as S) for the entire recording, as well as during the dark/light phases. Additionally, a COUNTIF function was used to count the number of REM events with a z-score EMG higher than 2 standard deviations (SD), which were further defined as RBD-like events or REM events without atonia. For each animal, the number of RBD-like events, as well as the ratio of RBD-like events to total REM events, was further extracted. Furthermore, for each sleep, awake, and REM event, the average PB value for each band-pass frequency was extracted and subjected to further comparisons. See: 10.17504/protocols.io.81wgbxjdolpk/v1.

### Fiber photometry during sleep

A subset of mice from each group received a combination of two adeno-associated viruses (AAVs) at a ratio of 1:1 for imaging dopamine release (AAV9-hSyn-GRAB-rDA1h, 250 nL, Addgene #140557) or acetylcholine release (AAV1-CAG-iAChSnFr, 250 nL, Addgene #137955) in the dorsomedial striatum (coordinates from the Bregma: AP: 0.5 mm, ML: 1.1 mm, DV: 3.0 mm) in the same injection. After a 2-week interval, mice were implanted with a sleep recording telemetry device and an optic fiber (0.50 NA, 400 µm, 4-mm long, Thorlabs) above the virus injection site. The ceramic ferrule holding the optic fiber was secured in place with dental cement. Following recovery, animals were acclimated to the sleep recording room for 48 h. During acclimation, mice were connected daily to the fiber photometry setup for habituation.

The fiber photometry setup enabled excitation of the isosbestic channel (405 nm), GCaMP (460–490 nm), and RCaMP (555–570 nm), with fluorescence collection at 500–540 and 580–680 nm (Built-in detector Gen 2 6-port Fluorescence mini cube, Doric lenses). Excitation was controlled using the Fiber Photometry console and analyzed through the manufacturer software (Doric Neuroscience Studio v2.0). All three excitation wavelengths were used in a lock-in protocol with non-overlapping frequencies, and the excitation LED power was set at 10% below the level that provided clear calcium events. This adjustment aimed to prevent arbitrary calcium release that could reduce signal variation in large windows (20 s). The setup allowed simultaneous collection of isosbestic, Ach-SnFr, and rGRAB-DA signals at 1017 Hz. A TTL input synchronized photometry and sleep signals. For each animal, a minimum of 30 min of data were acquired and repeated multiple times to cover multiple sleep cycles. Recordings were limited to 30 min to prevent the excitation light from affecting the sleep schedule of mice and to minimize interference from the patch cord on their ability to sleep.

Following the recording, sleep scoring was processed as described above, extracting timestamps of Wake, REM, NREM, and RBD-like events. Using a custom-made Matlab script, photometry data were downsampled to 500 Hz (using average smooth window function) and then aligned with the sleep scoring. For each channel, the signal at a specific time (F) was normalized to the average signal (F0) over a moving window of 5 s centered on the same point to obtain the normalized F/F0 signal. For each 1-min window, each channel of photometry was normalized using the z-score function and then averaged over the 20-s window corresponding to the sleep scoring. For each animal, photometry data were then averaged for specific sleep stages and compared across groups. See: 10.17504/protocols.io.6qpvr8xw3lmk/v1.

### Tissue processing

After behavioral and physiological experiments, all animals underwent transcardial perfusion. The perfusion process commenced with the infusion of 20 mL of PBS 1×, followed by 20 mL of 4% paraformaldehyde (PFA), and finally, 5 mL of PBS 1× to mitigate autofluorescent artifacts associated with PFA. After perfusion, various tissues, including the brain, spinal cord, kidney, liver, lungs, eyes, heart, stomach, intestine, and additional skin from the thoracic region, were harvested and immersed in 4% PFA for post-fixation, a duration not exceeding 4–6 h. After post-fixation, all tissues were individually segregated and transferred to a 30% sucrose solution for a period of 2–3 days. Thereafter, the tissues were transitioned to a cryoprotective solution (comprising 50% PBS 10×, 30% ethylene glycol, and 20% glycerol) for storage at − 20 °C until further processing. During the processing phase, all tissues were gently dried and subsequently embedded in an optimal cutting temperature (OCT) compound prior to sectioning at a thickness of 40 µm using a cryostat. See: 10.17504/protocols.io.bp2l622zzgqe/v1.

### Immunohistochemistry—antibody list

For fluorescent staining, primary antibodies for the following proteins were used: TH (raised in rabbits, 1:1000, AB152, Sigma-Aldrich), TH (raised in chickens, 1:1000, AB76442, Abcam, Cambridge, United Kingdom), choline acetyltransferase (ChAT, raised in goats, 1:1000, AB144P, Abcam), αSyn phosphorylated at serine 129 (pS129-αSyn, raised in rabbits, 1:500, AB51253, Abcam), apolipoprotein E (ApoE, raised in goats, 1:500, 178479, Millipore).

Secondary antibodies were as follows: Alexa 488-Anti rabbit (raised in donkeys, 1:1000, A2206, Jackson Immunoresearch, West Grove, Pennsylvania, PA); CY3-Anti rabbit (raised in goats, 1:1000, A11011, Thermofisher, Whaltham, MA); CY5-Anti rabbit (raised in goats, 1:1000, A21244, Thermofisher); Alexa 488-Anti chicken (raised in goats, 1:1000, A11039, Thermofisher); CY3-Anti chicken (raised in donkeys, 1:1000, A78951, Jackson Immunoresearch); CY5-Anti chicken (raised in donkeys, 1:1000, A21449, Jackson Immunoresearch); CY3-Anti goat (raised in donkeys, 1:1000, A11058, Jackson Immunoresearch); CY5-Anti goat (raised in donkeys, 1:1000, A214447, Jackson Immunoresearch); Alexa 488-Anti mouse (raised in donkeys, 1:1000, a21202, Thermofisher); CY3-Anti mouse (raised in donkeys, 1:1000, 715-175-150, Jackson Immunoresearch); CY5-Anti mouse (raised in donkeys, 1:1000, 715-165-150, Jackson Immunoresearch); Biotin-conjugated goat Anti-rabbit (1:500, B-6648, Sigma Aldrich).

### Immunohistochemistry—fluorescent

Freshly sectioned tissue slices were first washed 2–3 times with 1× PBS to remove OCT. Next, sections were blocked with 5% normal donkey/goat serum in 0.3% Triton X-100 in 1 × PBS for 1 h at room temperature before washing 5 times in 1× PBS. Following washes, all sections were transferred to a primary antibody solution containing a mix of primary antibodies diluted in 0.3% Triton X-100 in 1× PBS with 1% normal donkey/goat serum overnight at 4 °C. The following day, sections were washed 5 times in 1× PBS before being transferred to a secondary solution containing the adequate secondary antibody diluted in 0.3% Triton X-100 in 1× PBS with 1% normal donkey/goat serum for 4 h at room temperature. Following staining, sections were washed 3 times in 1× PBS before being mounted on microscope slides with mounting medium (Vectashield Mounting Medium with 4',6-diamidino-2-phenylindole (DAPI), H-1800-10, Vector Labs, Newark, NJ). To avoid variability in staining linked with the protocol, all animals from the same experiments (Gut injection, AAV-αSyn or αSyn-PFF injection in SNc/STR) were stained at the same time with their respective controls. See: 10.17504/protocols.io.j8nlk88y6l5r/v1**.**

### Immunohistochemistry—DAB

Freshly sectioned tissue slices were first washed 2–3 times with 1× PBS to remove OCT. Next, sections were quenched for 15 min in 3 mL of quenching solution (0.1 mL 30% H_2_O_2_, 0.1 mL methanol and 0.8 mL 1× PBS), then washed 4–5 times in 1× PBS. Next, sections were blocked and stained with primary antibody similarly to fluorescent staining (pS129-αSyn primary antibody, 1:500) overnight at 4 °C. The following day, sections were washed 4–5 times with 1× PBS and transferred into a secondary solution for 2 h at room temperature. Subsequently, sections were washed 4–5 times in 1× PBS and transferred into ABC Kit solution (PK4000, Vector Laboratories, Newark, NJ) containing 10 µL of solution A and 10 µL of solution B for 1 mL of 1× PBS. Sections were incubated for 1 h at room temperature with ABC Kit solution and quickly washed 4–5 times in 1 × PBS. Finally, sections were transferred to the DAB working solution (SK-4100, Vector Laboratories) under a fume hood. The 24-well-plate containing sections were gently shaken during staining and the reaction was stopped with transfer to 1× PBS based on the appearance of a dark signal intensity in a subset of sections (i.e. the limiting incubation factor is appearance of strong pS129-αSyn staining in a group). Sections were then washed 3–5 times in 1× PBS and quickly mounted on microscope slides. Following drying overnight at room temperature, slides were then transferred to sequential baths of distilled water (~ 2 min), 70% ethanol (2 times, ~ 2 min), 95% ethanol (2 times, ~ 2 min), 100% ethanol (2 times, ~ 2 min) and then 10% xylene (2 times ~ 5 min) to allow section dehydration. Following the last bath, sections were dried at room temperature (~ 5 min) and covered with DPX mounting medium. To avoid variability in staining linked with the protocol, all animals from the same experiments (Gut injection, AAV-αSyn or αSyn-PFF injection in SNc/STR) were stained at the same time with their respective controls. See: 10.17504/protocols.io.n2bvj34zplk5/v1.

### Staining—CongoRed

Freshly sectioned tissue slices were first washed 2–3 times with 1× PBS to remove OCT. Subsequently, all sections were mounted on microscope slides and left to dry at room temperature overnight. Next, slides were transferred into a 1% CongoRed solution for 30 min, followed by an alkaline bath (1% sodium hydroxide in 50% ethanol) for 5 min. Slides were then transferred to 3 sequential baths of water to remove excess staining. Slides were then transferred into 0.4% Toluidine blue solution for 10 min before being washed in 3 sequential baths of water to remove excess staining. Finally, the slides were dehydrated using sequential baths of distilled water (~ 2 min), 70% ethanol (2 times, ~ 2 min), 95% ethanol (2 times, ~ 2 min), 100% ethanol (2 times, ~ 2 min), and then 10% xylene (2 times, ~ 5 min). Following the last bath, sections were dried at room temperature (~ 5 min) and covered with DPX mounting medium. To avoid variability in staining linked with the protocol, all animals from the same experiments (gut injection, AAV-αSyn or αSyn-PFF injection in SNc/STR) were stained at the same time with their respective controls. See: 10.17504/protocols.io.4r3l22k1jl1y/v1.

### Imaging—confocal microscopy

Sections were observed by confocal microscopy (Carl Zeiss LSM 880, Oberkochen, Germany). All sections were captured at 10× magnification in air, featuring a resolution of either 1024 × 1024 or 2048 × 2048 pixels for high-resolution images. Tile scans were subjected to processing with a 0.6 zoom factor and 10% overlap for automated reconstruction. Z-stack imaging was performed with 1–4 µm spacing, and stack projection was executed using ImageJ (https://imagej.net/RRID:SCR_003070), excluding approximately 10% of the section's surface. Subsequently, all sections underwent processing in ImageJ software, incorporating consistent steps such as overlapping adjustments, cropping, color correction, and attribution. It is noteworthy that the post-processing of images for sections within the same experiment, specifically those involving gut injections, AAV-αSyn, or αSyn-PFF injection in the SNc/striatum, adhered to identical protocols and parameters. See: 10.17504/protocols.io.j8nlk88y1l5r/v1.

### Imaging—bright field

The mounted and covered sections were imaged with a NanoZoomer slide scanner (Hamamatsu, Shizuoka, Japan) with a 40× magnification in air. All sections were extracted using the NDP.view software and saved as TIFF files. See: 10.17504/protocols.io.rm7vzjj9rlx1/v1.

### Image processing—whole-brain DAB

TIF files extracted from the NDP.view software were imported into ImageJ for further analysis. Sections corresponding to distinct brain structures, such as the prefrontal cortex, striatum, GPe, thalamus, SNc, pons, cerebellum, and dorsal motor nuclei of the vagus nerve (DMV), were merged, cropped, and aligned within ImageJ. To prevent alterations in pixel size, adjustments were made in both the x and y directions. Only complete sections (except for the cerebellum, where most lobes are folded or damaged) were included in subsequent analyses. After positioning each section accurately, files were saved in designated folders for subsequent analyses. Utilizing a custom Matlab script, images from specific folders were opened, converted to an 8-bit grayscale (0–255 scale), and normalized to the signal across the entire image. This normalization process aimed to reduce signal variations associated with the transition between background and brain tissue.

To facilitate adequate structural overlapping, pixels were downsampled using a 5 × 5 filter, enabling the definition of 30 µm^2^ size for the regions of interests (ROIs). For each section within a specific group (monomeric αSyn, 2 weeks, 1 month, 3 months, 6 months, or 7 months), specific ROIs were then averaged for density plots and compared between groups using a one-way ANOVA. Post-hoc analyses were subsequently conducted for significant ROIs displaying group effects. The resulting images were plotted with a consistent scale bar using the images – Jet Matlab function. See: 10.17504/protocols.io.n2bvj34zplk5/v1.

### Cell counting

Following immunostaining for TH, ChAT, and DAPI, 2–3 sections encompassing the SNc/Ventral tegmental area (VTA), DMV, or laterodorsal tegmental area (LDT) were collected for high-resolution scanning (2048 × 2048 pixels) utilizing tile scanning and z-stack acquisition. Subsequently, images were imported into ImageJ, subjected to average projection, and color-adjusted to facilitate the differentiation of distinct anatomical structures.

For each section, the quantification of TH/ChAT/DAPI-positive neurons was manually performed using the multi-selection tool in Image J, and the counting area's surface was determined using the selection tool in Image J. The numbers of cells in the SNc, VTA, or DMV were then normalized by the surface area to derive the relative density.

Regarding ApoE quantification, 3–4 high-magnification images were acquired for each section within the SNc for each animal. Within the SNc region delineated by TH staining, the manual counting involved TH-positive neurons, ApoE puncta, and TH-positive neurons with at least one ApoE punctum within the cell. See: 10.17504/protocols.io.14egn66rql5d/v1.

### Cluster counting

Structures stained for pS129-αSyn were subjected to high-resolution scanning with a pixel size of 6.25 µm^2^. Subsequently, the images were imported into ImageJ, converted to grayscale, and the signal intensity was adjusted to a standardized threshold of 95% (consistent thresholds, exposure settings, and laser intensities were applied across all scans). Following this step, the "Process – Binary – Convert to Mask" function was employed. The mask function was determined using the default method against a black background. Next, a watershed filter was applied to select clusters with similar distribution. Finally, the "Analyze Particles" function was executed with a size range of 0 to infinity, displaying and summarizing the results. This facilitated the extraction of all clusters, including their coordinates and areas.

The subsequent step involved extracting clusters with a size ≤ 1 pixel (6.25 µm^2^) and clusters identified as artifacts (> 15,000 µm^2^), yielding the total number of clusters. Utilizing the Excel COUNTIF function, the numbers of small clusters (6.25–50 pixels), medium-sized clusters (50–200 pixels), and large clusters (> 200 pixels) were calculated as proxies for the size of pS129-αSyn aggregates.

Following the extraction of tissue surface and cluster count data, their relative density was determined for further analysis. Automated ImageJ macros were employed for the same procedures across all tissues (peripheral and brain) and animals, ensuring consistency in the analytical process. See: 10.17504/protocols.io.5jyl8224rl2w/v1.

### Data exclusion

Outliers were identified using the ROUT method (Q = 5%) in GraphPad Prism (version 9; RRID:SCR_000306). For the behavioral experiment with the operant boxes, animals were excluded if they did not proceed to a correct poke over the entire 24-h period; however, the animals were still tested for the next protocol. Only animals that did not execute the correct poke for 2 consecutive sessions were excluded. For sleep experiments, data with more than 1% of artifacts detected were automatically excluded. For sleep electrophysiology experiments, 20-s events that were considered as artifacts by the software were automatically discarded from analyses, but the animals were maintained for further analyses. For RBD-like events, animals that displayed no REM events during the entire recording were automatically discarded from further analyses. For virus experiments (AAV-hSNCA or photometry), post-hoc histology of the virus location and expression was used for discarding animals with misplaced injections. For photometry experiments, all animals with a misplaced optic fiber or a signal that showed no variation compared to the isosbestic signal, were discarded from further analyses.

### Statistical analyses

All statistical analyses, raw values, and sample numbers are available on the zenodo.org platform following the 10.5281/zenodo.10822457. Statistical analyses were conducted using GraphPad Prism software (version 9). For comparisons involving two groups, paired or unpaired *t*-test analyses were applied. For comparisons involving three or more groups, Bartlett’s test was done to define differences in the standard deviation. In the case that Bartlett’s test was found not significant (*P* > 0.05), a one-way ANOVA analysis with a Tukey post-hoc analysis was performed. If Bartlett’s test was found to be significant (*P* < 0.05), a Brown-Forsythe and Welch ANOVA test was performed together with Dunnett’s test for post-hoc analyses. In the analyses of gut-injected animals, post-hoc analyses were provided for the comparison of monomeric αSyn-injected animals with the αSyn-PFF-injected group, with additional statistical values available in the Table S1. For comparison of the surgery effect (i.e. monomeric αSyn 2 weeks vs. 7 months) an unpaired *t*-test was conducted. For comparisons of sex differences, a two-way ANOVA sex × group analysis was conducted, and the interaction effect was reported in the figure legend. In anatomical analyses of ROIs, a one-way ANOVA or two-way ANOVA analysis was performed on each ROI and presented as images – Jet (Image J), while post-hoc analyses were conducted using Dunnett’s test by default. *P* < 0.05 was considered as statistically significant.

## Results

### Injection of αSyn-PFFs induces the formation of αSyn aggregates along the caudo-rostral axis of the CNS

**Fig. 1:**
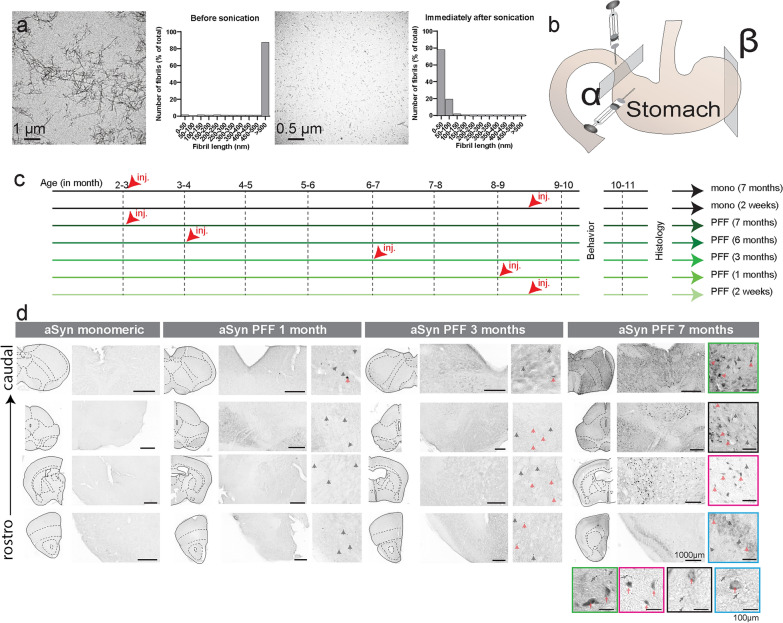
Propagation of alpha-synuclein aggregates in the periphery following injections of preformed fibrils in the stomach. **a** Transmission electron microscopy of fibrils before and after sonication revealed a change from long fibrils to small-sized fibrils. **b** Graphical representation of the injection sites in the muscularis layers of the duodenum and stomach in mice. α represents the transverse section at the level of the injection site, while β illustrates sections located on the distant side. **c** Experimental design. Age-matched animals were selected from litters born within the same period and were tested for behavior and electrophysiology concurrently. Each group, including monomeric-injected or PFF-injected animals, received a single injection at 4 different sites located within the stomach and duodenum. **d** High-resolution images and their respective inserts of brain sections stained for DAB–pSer129 from animals injected with monomeric αSyn or αSyn-PFFs in the stomach. The black arrows indicate neurite staining, whereas the red arrows indicate somatic expression of pSer129. The color code refers to the respective images on the horizontal panel

Sonication of recombinant mouse αSyn fibrils generated αSyn-PFFs [10], with the majority of fibrils measuring less than 50 nm in length (Fig. 1a). Injection of αSyn-PFFs into the muscularis layers of the duodenum and glandular stomach (Fig. 1b) for 1 month resulted in rapid diffusion of pS129-αSyn staining throughout the entire stomach, including the forestomach (Fig. S1). To assess the temporal diffusion in the periphery and central nervous systems, 71 animals were injected once with either monomeric αSyn (2 weeks before behavior tests *n* = 7, and 7 months before behavior tests *n* = 9 mice, 4 additional animals were injected with αSyn mono for 2 weeks and added to sleep recording experiments) or αSyn-PFF at different time points (2 weeks* n* = 6, 1 month *n* = 8, 3 months *n* = 10, 6 months *n* = 21 and 7 months *n* = 10 mice). All mice were wild-type (WT) on the C57BL/6 background of the same age at the time of the behavior experiments and were tested together to minimize group variability (Fig. 1c). Following behavioral assessments, animals were sacrificed, and tissues were collected. For all histology and behavioral experiments, data from monomeric αSyn-injected mice at 2 weeks and 7 months before being sacrificed were compared (Fig. S2–S3). Since no differences on anatomical and behavioral parameters between the time points were found, data were combined.

Aggregated αSyn has been reported in biopsies of the gastrointestinal tract from PD patients [13, 14]. In the brain, following DAB-staining of pSer129, we found the presence of low-signals in monomeric injected animals similar to previous observations (Fig. 1d) [15, 16]. In sharp contrast, in animals injected with αSyn-PFF, we found a progressive spread of the pathology along the caudo-rostral axis with the presence of neuritic staining (black arrows) as well as somatic (including nucleus and cytoplasm) staining (red arrows) that propagates in the pons, midbrain and forebrain at different time points following injection (Fig. 1d). It is worth noting that all brain sections were processed at the same time and using the exact same protocol, suggesting that the observed differences between monomeric and αSyn-PFF-injected animals were indeed a specific signal rather than an artifact. This was confirmed by the similarity of the signal to previous observations in control animals [15].

Altogether, our data suggest that injection of αSyn-PFF in the stomach/duodenum results in αSyn aggregates that increase in size over time and spread along the caudo-rostral axis.

### Time-dependent propagation of pathological αSyn in the CNS

**Fig. 2:**
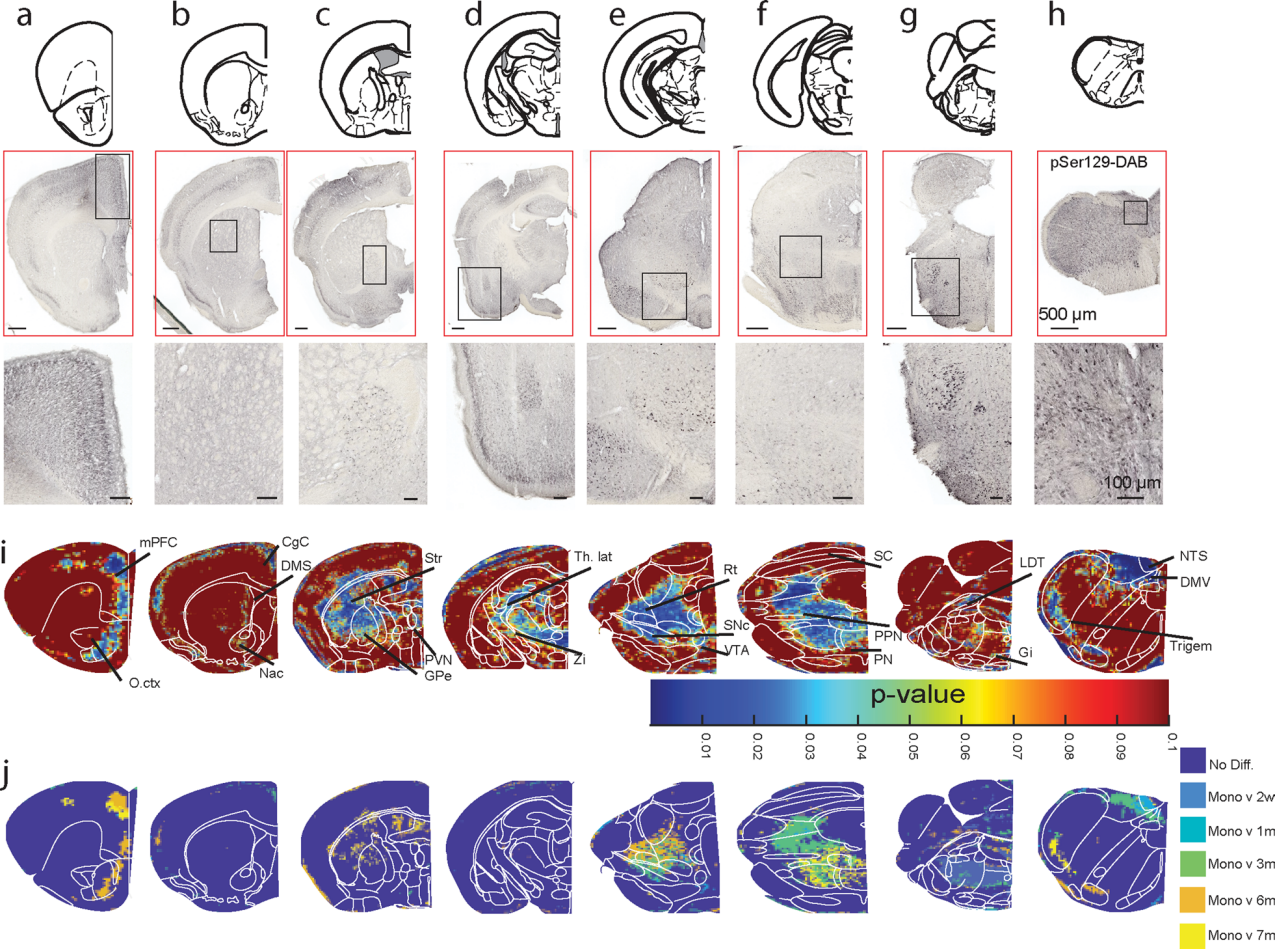
Time-dependent propagation of pS129-αSyn in the brain. **a–h** Whole-brain mapping of the temporal propagation of pS129-αSyn DAB staining in **a** frontal cortex, **b** striatum, **c** forebrain, **d** thalamus, **e** midbrain, **f** brainstem, **g** pons and **h** medulla. DAB images shown here were obtained from the same animal injected with αSyn-PFFs in the stomach for six months. Density plots are estimated on 30-µm^2^ ROIs for each animal. **i** Color-coded *P*-value was obtained from the “ROI-by-ROI” comparison between monomeric αSyn and αSyn-PFF groups using one-way ANOVA. **j** Post-hoc analyses were obtained using multiple comparisons to the monomeric αSyn group. Statistical data represent post hoc analyses compared to the monomeric αSyn group following one-way ANOVA

According to Braak’s model of αSyn propagation, the formation of pathologic αSyn in the gut will propagate to the brain along the vagus nerve and induce non-motor phenotypes based on the structures impacted at each stage [7, 17]. As the aggregates found in the DAB-staining of pSer129 were dense when focusing on specific brain structures, we used an automatic mapping of pS129-αSyn staining at the whole brain level (Figs. 2 and S2).

After scanning brain sections stained for pS129-αSyn at high resolution, we cropped and aligned the different sections before proceeding to normalize density plots and statistical analyses on 30 µm^2^ ROIs (Fig. S2a, b) [18]. Here, background normalization of the signal was done by using the signal on the entire brain slice and thus normalizing ROIs to the background signal (Fig. S2a). Analyses were done by comparing group effects in each ROI (Fig. 2i) and using a post hoc test to define the time points when pS129-αSyn intensities increase and remain significantly different for αSyn-PFF-injected animals compared with monomeric αSyn-injected animals (Fig. 2j). The outline of key brain structures was done using the Paxinos Brain Atlas [19] to compare pS129-αSyn intensity in brain regions (Table S1). First, we compared pS129-αSyn intensity between animals with a single monomeric injection at 2 weeks and 7 months before behavior test and found no significant differences between these groups (Fig. S2c–j). Injection of αSyn-PFF in the gut resulted in an early increase of pS129-αSyn in ROIs located in the DMV (~ 2 weeks to 1 month, Fig. 2h) followed by the gigantocellular nuclei (Gi, ~ 1 month, Fig. 2h), nucleus of the solitary tract (NTS, ~ 1 month, Fig. 2h), the pons (~ 3 months, Fig. 2g), substantia nigra (SN, ~ 3 months, Fig. 2e), the globus pallidus (GPe)/striatum (~ 6 months, Fig. 2c) and cortical structures (~ 3–6 months, Fig. 2a–d). Despite the significant increase of pS129-αSyn in the thalamus (Fig. 2d), we were not able to define the exact timepoints at which the effect was significant. This could be due to the complexity of thalamus anatomy [20], but may be also related to a transient increase in pS129-αSyn signal. We also observed an increase in pS129-αSyn intensity in ROIs located in vestibular structures (Fig. 2g, h), including the trigeminal nuclei, but post hoc analyses suggest the effect was significant only in late stages (~ 6 months, Fig. 2j). We next measured pS129-αSyn intensity in specific nuclei and confirmed that the intensity was significantly increased in the entire DMV at 1 month and in the SNc at 3 months post-injection (Table S1).

**Fig. 3:**
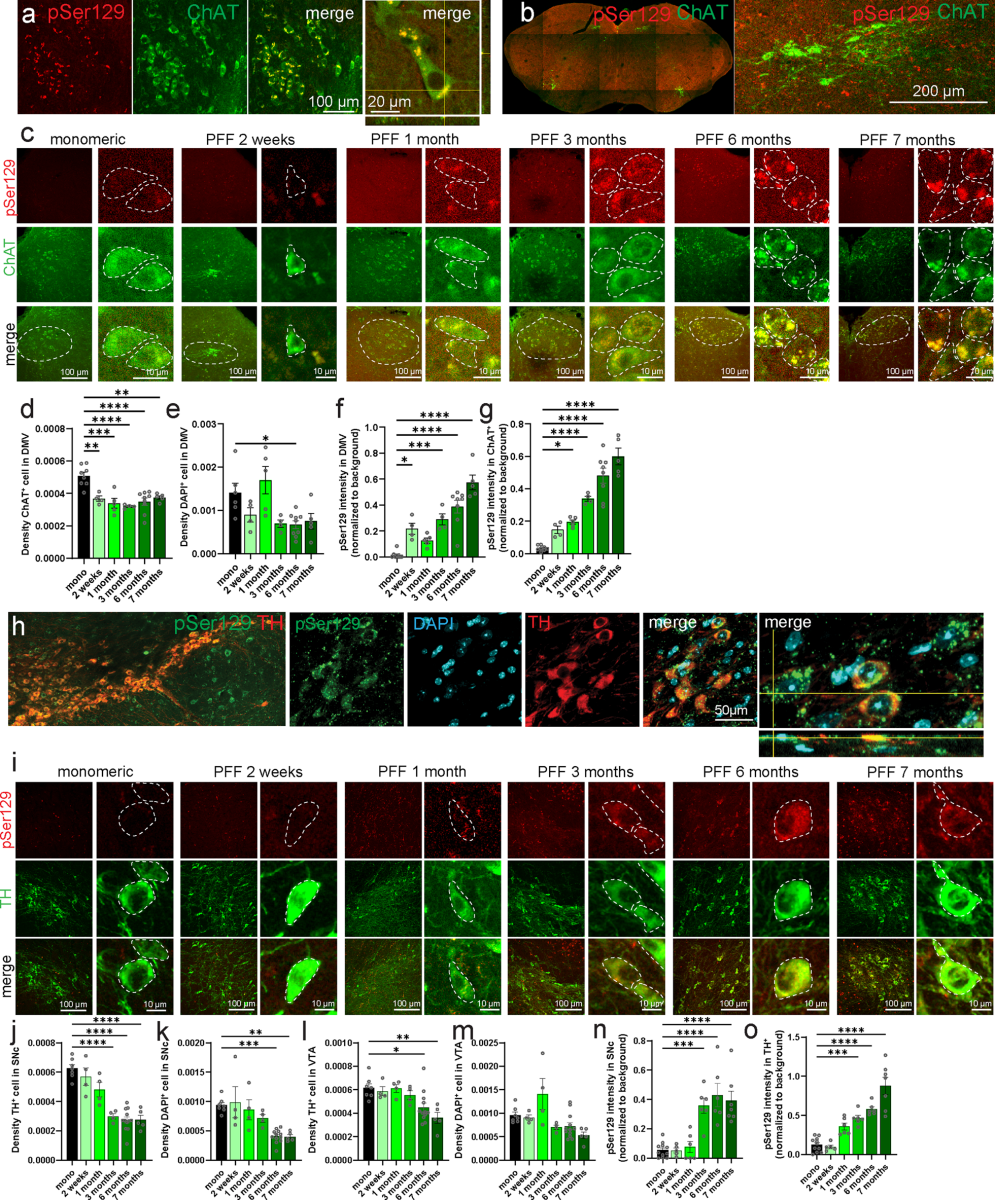
Time-dependent loss of neurons in the DMV and the SNc. **a–c** Confocal images of pS129-αSyn and ChAT staining in the DMV of mice injected with monomeric αSyn or αSyn-PFFs (**a**: 1 month αSyn-PFFs; **b**: 7 months αSyn-PFFs). In (**c**), the panels on the far left are representative images of pSer129 staining in the DMV of an animal injected with monomeric αSyn and the corresponding high-resolution insert. From left to right, the images show animals injected with αSyn-PFFs in the stomach 2 weeks, 1 month, 3 months, 6 months, and 7 months prior to the staining, respectively. **d–g** Quantification of the density of ChAT-positive neurons (**d**), density of DAPI-positive neurons in the DMV (**e**), pSer129 intensity in the entire DMV (**f**), and pSer129 intensity in individual ChAT-positive neurons in the DMV **(g)** of mice injected with monomeric αSyn or αSyn-PFF. **h, i** Confocal images of pS129-αSyn and TH staining in the SNc of mice injected with monomeric αSyn or αSyn-PFF. **j–o** Quantification of the density of TH-positive neurons in the SNc (**j**), density of DAPI-positive neurons in the SNc (**k**), density of TH-positive neurons in the VTA (**l**), density of DAPI-positive neurons in the VTA (**m**), pSer129 intensity in the entire SNc (**n**), and pSer129 intensity in individual TH-positive neurons in the SNc (**o**) of mice injected with monomeric αSyn or αSyn-PFF. Data are expressed as mean ± SEM. Each point represents an individual animal. *P < 0.05, **P < 0.01, ***P < 0.001, ****P < 0.0001. Statistical data represent post-hoc analyses compared to the monomeric αSyn group following one-way ANOVA. **c, i** Inserts showing high-resolution (63×) images of individual cells with borders delimited by white lines. Images were acquired with the same confocal properties and presented with the same contrast

To determine the intracellular distribution of pS129-αSyn in the pons and midbrain, we performed immunofluorescent labeling using markers for cell types that are reduced in PD including TH and ChAT (Fig. 3) [21]. Similar to DAB-staining, all sections were processed at the same time using the same protocols and images were always acquired using the same confocal parameters. In agreement with previous reports, immunostaining of pS129-αSyn revealed a basal level in monomeric injected animals in the DMV and the SNc, but the signal increased following injection of αSyn-PFF in the stomach (Fig. 3) and appeared similar to the staining reported before [15, 22].

In the DMV (Fig. 3a–c), staining for pS129-αSyn and ChAT revealed a decrease of the number of ChAT^+^ neurons as early as 1 month post-injection (Fig. 3d, e). In addition, we observed an increase of the overall pS129-αSyn intensity in the entire DMV (Fig. 3f) as well as individual neurons (Fig. 3g) starting ~ 1–3 months following αSyn-PFF injection. These data resembled the data in the 6-OHDA-lesioning model of parkinsonism [23].

Similarly, in the midbrain, pS129-αSyn was observed mostly in the soma of neurons, including TH^+^ and TH^−^ cells (Fig. 3h, i), and the intensity within individual neurons increased in animals injected with αSyn-PFF compared to monomeric αSyn-injected mice. In the SNc, we found a reduction of TH^+^ neurons at 3 months post-injection (Fig. 3j), while the reduction in DAPI^+^ cells occurred later at 6 months (Fig. 3k). Neurons in the neighboring VTA presented a decrease of TH^+^ neurons around 6 months post-injection (Fig. 3l), but no significant reduction of DAPI (Fig. 3m). In addition, we found increases from 3 months post-injection of the overall pS129-αSyn intensity in the SNc (Fig. 3n) as well as the average intensity per individual neurons (Fig. 3o).

### Surgery does not have effects on the non-motor or motor functions

To examine whether the phenotypic alterations could be linked to recovery from surgery rather than to the αSyn-PFF injection itself, we compared animals injected with monomeric αSyn 2 weeks versus 7 months before testing (Fig. S3). We did not observe significant differences between the mice injected with monomeric αSyn at the two timepoints in any non-motor or motor manifestations, including sleep, depression or locomotion. We therefore pooled both groups together for all analyses (Fig. S3, Table S2).

### Time-dependent development of non-motor symptoms related to PD

**Fig. 4:**
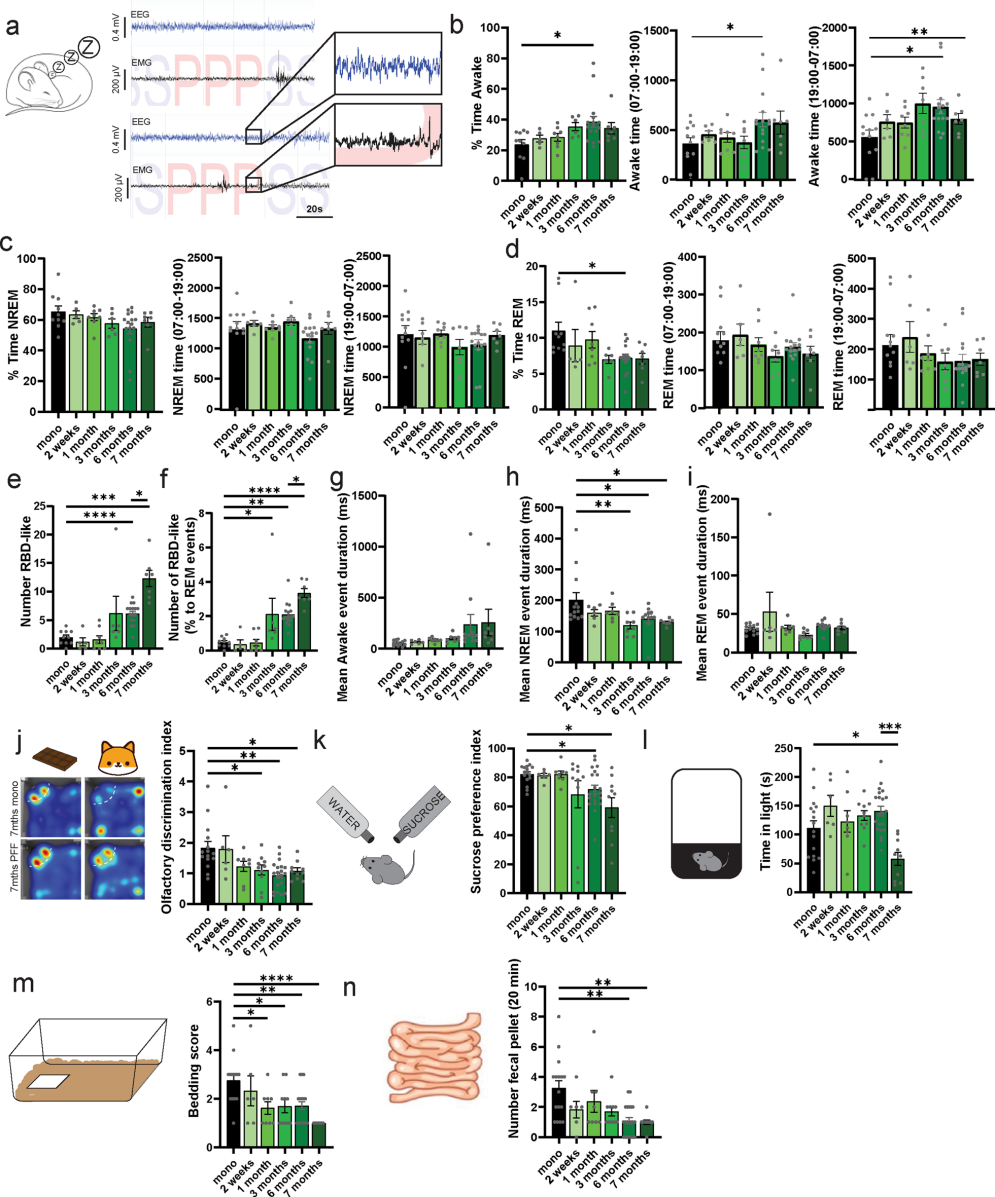
Time-dependent development of non-motor phenotypes following injection of PFF in the stomach. **a** Example of EEG (blue) and EMG (black) signals during NREM (S) or REM (P) sleep. The top panel represents muscle contraction during NREM sleep, while the bottom panel represents REM sleep behavior disorder (RBD-like). **b–d** Percentage of time spent awake (**b)**, in NREM (**c)**, or in REM (**d**) following injection of monomeric αSyn or αSyn-PFF, during the entire day, light phase, and dark phase. **e** Number of RBD-like events per 24 h in mice injected with monomeric αSyn or αSyn-PFF. **f** Number of RBD-like events normalized to the individual number of REM events in mice injected with monomeric αSyn or αSyn-PFF. **g–i** Average duration of awake (**g**), NREM (**h**), or REM (**i**) events following injection of monomeric αSyn or αSyn-PFF. **j** Olfactory discrimination index expressed as the ratio of time spent in the corner associated with chocolate or Trimethylthiazoline odors in mice injected with monomeric αSyn or αSyn-PFF. **k** Sucrose preference index expressed as the ratio of water to sugar-water consumed after 24 h in mice injected with monomeric αSyn or αSyn-PFF. **l** Time spent in the light area of the dark–light test in mice injected with monomeric αSyn or αSyn-PFF. **m** Scoring on the bedding test (min) in mice injected with monomeric αSyn or αSyn-PFF. **n** Number of fecal pellets produced in 20 min in mice injected with monomeric αSyn or αSyn-PFF. Data are expressed as mean ± SEM. Each point represents an individual animal. *P < 0.05, **P < 0.01, ***P < 0.001, ****P < 0.0001. Statistical data represent post-hoc analyses compared to the monomeric αSyn group following one-way ANOVA. When significant, post-hoc analyses comparing six-month and seven-month PFF-injected animals are provided

To characterize if specific phenotypes, typically observed early in PD (i.e. mainly non-motor symptoms), occur following the injection of αSyn-PFF in the stomach, we tested mice for specific behaviors resembling non-motor symptoms in PD patients. Two of the main non-motor symptoms, which initially appear in the premotor and early stages of the disease and correlate with highly increased risk of PD, are shortened REM and RBD [24]. To assess to what extent αSyn-PFF injection into the stomach could impair sleep, we implanted the animals (monomeric *n* = 10, PFF 2 weeks *n* = 5, 1 month *n* = 8, 3 months *n* = 6, 6 months *n* = 16, 7 months *n* = 7 mice) with a subcutaneous telemetry device collecting frontal EEG and neck-muscles EMG. Automated sleep scoring allowed us to define the different stages of sleep, including awake, REM, and NREM. Using significant (> 2SD) muscle contraction in the root mean square of the EMG signal during REM events, we determined the number of events without atonia and defined them as RBD-like (Fig. 4a–i).

We found that a single injection of αSyn-PFF in the stomach did not overall impact sleep significantly, with a small increase in awake time during the dark phase (Fig. 4b) and a reduction in REM time during the entire day (Fig. 4d). However, the number of RBD-like events as well as the percentage of REM events with RBD-like activity increased significantly starting at 3 months post injection, and worsened with time (Fig. 4e, f). The injection of αSyn-PFF in the stomach did not impact awake or REM event duration, but decreased NREM event duration (Fig. 4g–i).

Interestingly, when analyzing cortical oscillations during RBD-like events or individual sleep stages, we found very little impact of αSyn-PFF injection on cortical oscillations, with an exception for an increase in theta oscillations during RBD-like events (Fig. S4), confirming that the RBD-like phenotypes might precede other sleep pathologies [25]. These data suggest that RBD-like events appear simultaneously with the increase of pS129-αSyn in the pons and the midbrain, including structures that have been associated with sleep regulation such as the LDT and the SNc.

In addition to sleep disturbances, hyposmia is correlated with the risk of developing PD [26]. To test olfaction in our mice, we performed an adjusted version of the olfactory discrimination test [27], where mice were successively exposed to chocolate or TMT (a chemical component of fox urine and feces) in a classical open field arena (Fig. 4j, S5a–j). This test allowed us to assess olfactory preference for emotionally positive or negative odors. First, we found that all animals independently of the injection of αSyn-PFF or their timepoints were able to show preference for the side paired with the chocolate smell, suggesting that olfaction was conserved (Fig. S5a–e). However, the αSyn-PFF-injected mice presented an impairment in the discrimination of TMT as early as 3 months following injection (Fig. 4j, S5f–j) and an abnormal increase in activity in the olfactory test as early as 1 month post-injection (Fig. S5a). The hyposmia-like phenotype at around 3 months after αSyn-PFF injection coincides with a period where pS129-αSyn increases in the parabigeminal nucleus, the dorsal raphe and the SNc. These structures are involved in innate behaviors and sensory processing [28] and show correlations with hyposmia in PD patients [29]. We next tested whether the injection of αSyn-PFF could induce a depression-like phenotype using the sucrose-preference test (Fig. 4k, S5k–m). We found a reduction of the sucrose-preference index in the late stage (~ 6 m) (Fig. 4k) that was due to a reduction in sucrose consumption rather than total intake (Fig. S5k–m). In addition, we found an increase in anxiety-like behavior using the dark–light box test in animals injected with αSyn-PFF 7 months before testing (Fig. 4l, S5n). We also observed a worsening in the nest bedding test confirming the depression-like phenotypes (Fig. 4m, S5o). Constipation is also one of the earliest premotor symptoms and a risk factor for PD [30]. We found a reduction in the number of fecal pellets expelled over 20 min in αSyn-PFF-injected mice compared to controls (Fig. 4n), with significant effects observed in animals injected 6 and 7 months before testing. This correlated with a reduction in total fecal weight (Fig. S5p), but not in average pellet weight (Fig. S5q). Drying the fecal pellet shows a reduction of the weight of the dry fecal pellet (Fig. S5r), but no effect on the water content (Fig. S5s), suggesting an impairment of intestine motility.

### Fine motor planning impairments

**Fig. 5:**
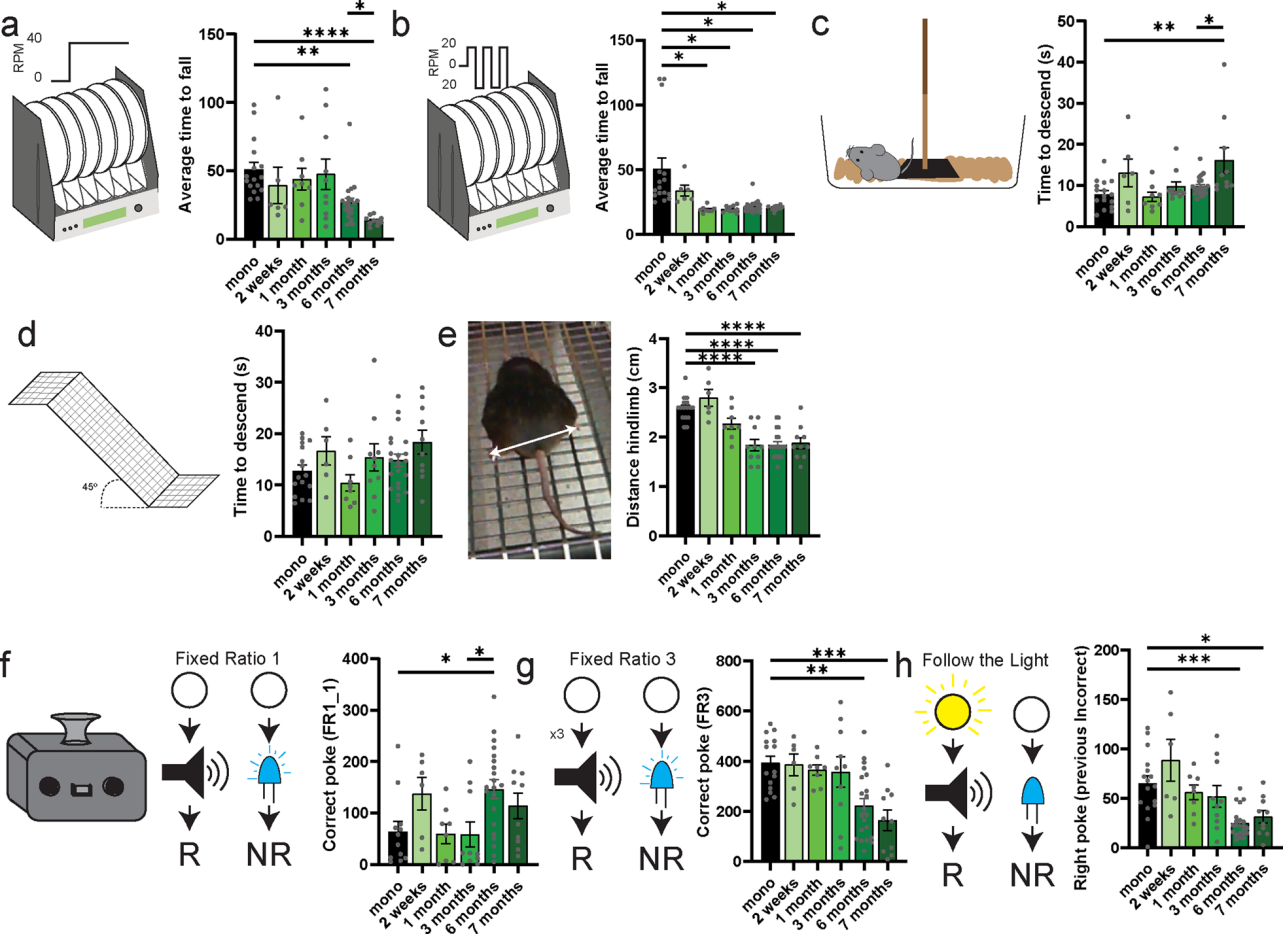
Development of locomotor symptoms following injection of αSyn-PFF in the stomach. **a, b** Latency to fall in the 40 RPM constant-speed (**a**) or the 20 RPM docking (**b**). **c** Time to descend the vertical pole for mice injected with monomeric αSyn or αSyn-PFF. **d** Time to descend the inclined platform for mice injected with monomeric αSyn or αSyn-PFF. **e** Average distance between hindlimbs during the inclined platform test in mice injected with monomeric αSyn or αSyn-PFF. **f–h** Number of correct pokes during the first session of fixed ratio 1 (**f**) or fixed ratio 3 (**g**), or during the subsequent light protocol (**h**) for mice injected with monomeric αSyn or αSyn-PFF. Data are expressed as mean ± SEM. Each point represents an individual animal. *P < 0.05, **P < 0.01, ***P < 0.001, ****P < 0.0001. Statistical data represent post-hoc analyses compared to the monomeric αSyn group following one-way ANOVA. When significant, post hoc analyses comparing six-month and seven-month PFF animals are provided

PD is characterized by specific motor symptomatology, including problems with gait initiation, turning, freezing of gait and postural control. Because many of these motor phenotypes are difficult to assess in classical models using conventional locomotor methods, we used an adapted version of the rotarod that prevents motor learning and allows characterization of various motor functions [31, 32]. Here, we tested motor symptoms in the high-speed (40 RPM) rotarod in untrained mice and found a reduction of the average time to fall across 3 sessions at the later stages in the αSyn-PFF injected mice (Fig. 5a), and the reduction progressively worsened across sessions in mice injected 6 months before testing (Fig. S6a–c). To reproduce the Time Up and Go test, a classic test employed to assess sequential locomotor tasks in PD, we used the docking setting at 20 RPM, again in untrained mice. In that protocol, the rotarod turns in a specific direction for 5 rotations before alternating toward the other direction. Interestingly, we found a significant reduction in the average time to fall across 3 sessions as early as 1 month post-injection (Fig. 5b) that was arising from the second session (Fig. S6d–f). This suggests that motor planning may be impaired at earlier stages before deficits in motor execution emerge. In comparison, testing the mice on the descending pole revealed an increased time to descend after 7 months post-injection (Fig. 5c), which was independent of the anxiety effect linked to the test (Fig. S6g–i). Similarly, testing the mice on the declined platform revealed no significant impairment of the time to descend (Fig. 5d), but a significant decrease of the distance between hindlimbs (i.e. clasping) as early as 3 months post-injection (Fig. 5e). Finally, when recording cortical activity separating the local field potential (LFP) during inactive and active periods, we found that animals injected with monomeric αSyn showed higher Delta (Fig. S6j–k), Theta (Fig. S6l–m), Alpha (Fig. S6n–o), and Sigma (Fig. S6p–q), but not Beta (Fig. S6r,s), oscillations during movement compared to immobile periods. In contrast, in mice injected with αSyn-PFF, the difference in Delta oscillation between movement and immobility was already lost at 1 month post-injection, and the difference for Theta was lost by 7 months post-injection, while Alpha, Beta, and Sigma oscillations did not show significant differences between movement and immobile states (Fig. S6j-s).

Altogether, these results suggest that motor planning might be impaired at an early stage, possibly when pS129-αSyn is present in the mesencephalic locomotor region, while motor function declines when dopaminergic degeneration becomes significant and affects basal ganglia circuitry [33, 34].

### Cognitive decline at later stages of the αSyn-PFF gut-brain model

In mid- to late-stage PD, cognitive flexibility is reduced and often progresses to dementia [35–37]. Here, we used in-home cage cognitive tasks [38] to test the ability of the mice to shift correctly between different cognitive paradigms. In the 3 sessions of Fixed-Ratio 1 (FR1), we found that mice injected with αSyn-PFF 6–7 months before testing displayed a longer time to associate the correct poke with reward delivery, but the mice still retained the ability to associate action with outcome (Fig. 5f, S7a–g). Interestingly, when the cognitive paradigm was modified by either changing the intra-dimensional states (FR3, Fig. 5g) or the extra-dimensional (follow the light, Fig. 5h), mice injected with αSyn-PFF for 6–7 months displayed a significant impairment of cognitive planning as determined by a reduction of the correct/incorrect poke ratio (Fig. S7h–l).

Altogether, these data suggest that cognitive planning is impaired in parallel with the propagation of pS129-αSyn to the striatum and the frontal cortex, two structures extensively linked with cognitive planning and set-shifting [39].

### Sex differences observed in motor behaviors

There are sex differences in symptom presentations between men and women with PD. For example, axial symptoms and cognitive decline are more common in men, whereas depression and insomnia are more common in women [40]. A cautionary note is that the sex effect in PD is influenced by menopause in women [41]. Nonetheless, to make an initial assessment of whether there is an influence of biological sex in the gut αSyn-PFF model, we first compared the propagation of pS129-αSyn as a function of sex by group (sex*group) (Fig. S8a-h). Interestingly, we found a few structures that did not show significant group effect (Fig. 2), but did present a sex*group interaction effect, with a faster propagation of pS129-αSyn in the motor cortex (Fig. S8a), striatum, medial septum (Fig. S8b) and the pons (Fig. S8g) in male mice. No effect was observed for other analyzed regions. We next compared the density of ChAT-positive neurons in the DMV (Fig. S8i) and TH-positive neurons in the SNc (Fig. S8j), but found no significant sex*group effects. Likewise, when we analyzed non-motor phenotypes (Fig. S8k-r), we found no significant sex*group effects on % time awake (Fig. S8k), % time in REM (Fig. S8m), normalized number of RBD-like events (Fig. S8n), olfactory discrimination (Fig. S8o), sucrose preference (Fig. S8p), time in the light zone of the dark–light box test (Fig. S8q) or the number of fecal pellets (Fig. S8r). We only observed significant sex*group effects in the mean awake event duration (Fig. S8l) and motor phenotypes, where males were affected earlier than females (Fig. S8s–u).

Altogether, these initial data show that some aspects of αSyn propagation and motor impairments may be influenced by sex in the studied gut-to-brain model. However, these data need to be interpreted cautiously and further characterization with larger group sizes is required.

### αSyn overexpression in SNc induces various sleep impairment phenotypes

**Fig. 6:**
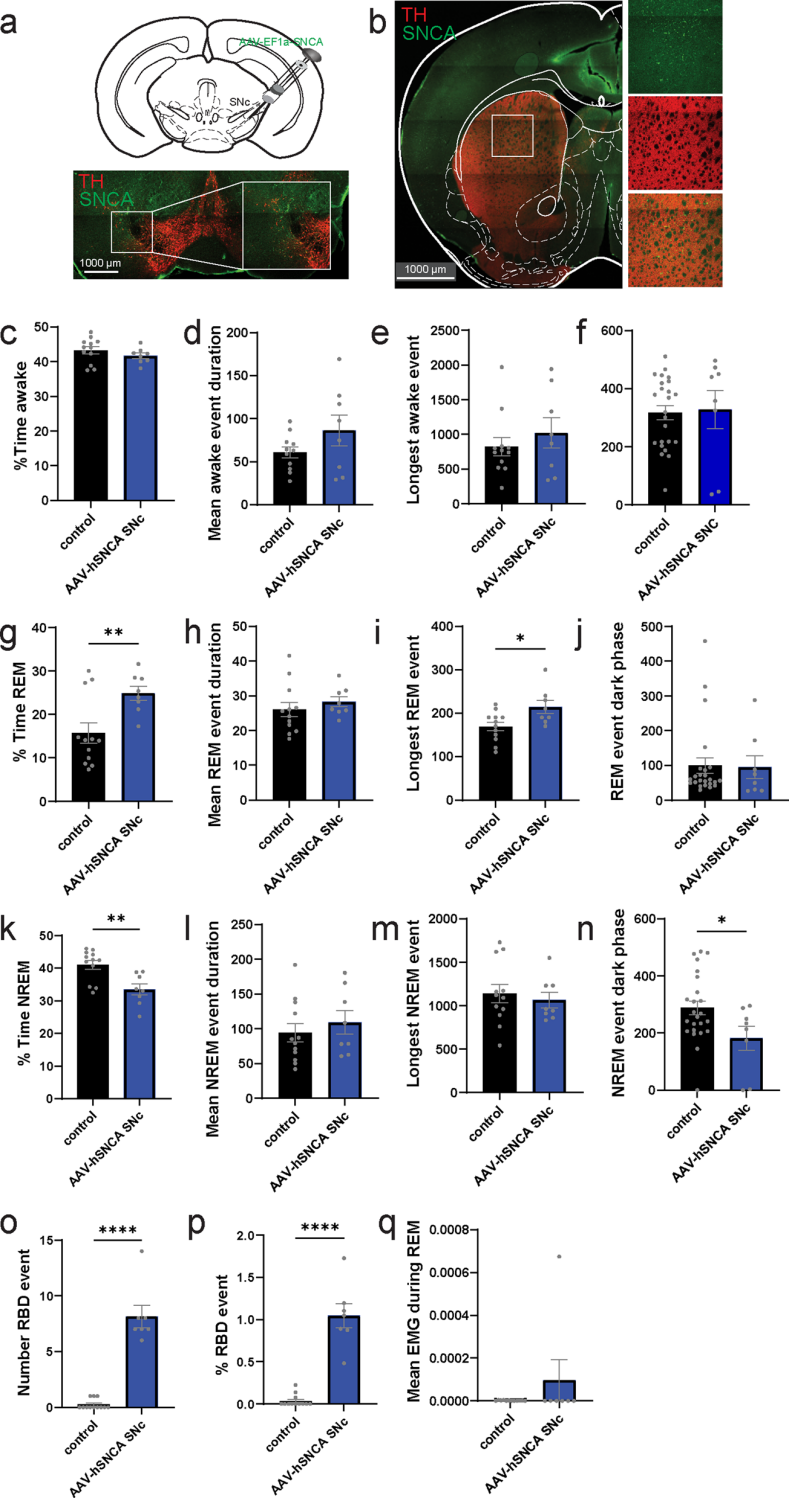
Sleep alteration with viral overexpression of alpha-synuclein in the SNc. **a, b** Confocal images of the injection of AAV-hSNCA in the SNc of WT mice showing expression of αSyn in the input structures of the SNc (**a**) and output (i.e., striatum, **b**), repeated for all mice. **c–f** Percentage of time awake (**c**), average awake event duration (**d**), duration of the longest awake event (**e**), and average time awake (during the dark phase) (**f**) during the 24-h recording in control mice (AAV-mCherry) or following injection of AAV-hSNCA in the SNc. **g–j** Percentage of time in REM (**g**), average REM event duration (**h**), duration of the longest REM event (**i**), and average time in REM (during the dark phase) (**j**) during the 24-h recording in control mice (AAV-mCherry) or following injection of AAV-hSNCA in the SNc. **k–n** Percentage of time in NREM (**k**), average NREM event duration (**l**), duration of the longest NREM event (**m**), and average time in NREM (during the dark phase) (**n**) during the 24-h recording in control mice (AAV-mCherry) or following injection of AAV-hSNCA in the SNc. **o, p** Number of RBD-like events (**o**) and percentage of RBD-like events over the number of REM events (**p**) during the 24-h recording in control mice (AAV-mCherry) or following injection of AAV-hSNCA in the SNc. **q** Average EMG signal during REM events during the 24-h recording in control mice (AAV-mCherry) or following injection of AAV-hSNCA in the SNc. Data are expressed as mean ± SEM. All individual points represent individual animals. *P < 0.05, **P < 0.01, ****P < 0.0001. Statistical data represent unpaired *t*-test

Following injection of αSyn-PFF in the stomach, we found sleep impairments, including an RBD-like phenotype, around 3 months post-injection (Fig. 3), which temporally paralleled the propagation of pS129-αSyn to the SNc (Fig. 2). In the clinic, a reduction of the dopamine transporter is observed in patients with idiopathic RBD [42, 43]. However, in our gut-brain model, prior to reaching the SNc, pS129-αSyn is observed in multiple structures that might be involved in sleep-related phenotypes. To confirm the involvement of dopamine neurodegeneration in RBD-resurgence, we specifically targeted the SNc using viral overexpression of *SNCA* as well as local injection of αSyn PFFs. To test whether αSyn expression in the SNc might impact the sleep pattern, we used viral overexpression of human αSyn (AAV-hSNCA, *n* = 8 mice) or control (AAV-mCherry, *n* = 12 mice) in the SNc (Fig. 6a) [44]. The overexpression of human αSyn (hαSyn) causes somatic, axonal and terminal expression (Fig. 6b). Following bilateral injections, mice were implanted with a telemetry head stage and sleep was scored 8 weeks post-injections. We found no alterations in parameters related to awake time following injection of AAV-hSNCA (Fig. 6c–f), but the injection increased the time in REM (Fig. 6g), without impacting the event pattern outside of the longest REM event (Fig. 6h–j). Not surprisingly, the increase of time in REM with viral overexpression of hαSyn in the SNc induced a reduction of the time in NREM (Fig. 6k-n). Finally, AAV-hSNCA increased the number of RBD-like events (Fig. 6o) and normalized RBD-like events (Fig. 6p), without generally affecting muscle activity (Fig. 6q).

Altogether, this suggests that viral overexpression of hαSyn in the SNc is sufficient to alter atonia during REM sleep (i.e. RBD-like signs) [45, 46], and suggests the possible involvement of dopaminergic neurons in this phenomenon.

### Different effects of somatic versus axonal propagation of αSyn-PFF in dopaminergic neurons

**Fig. 7:**
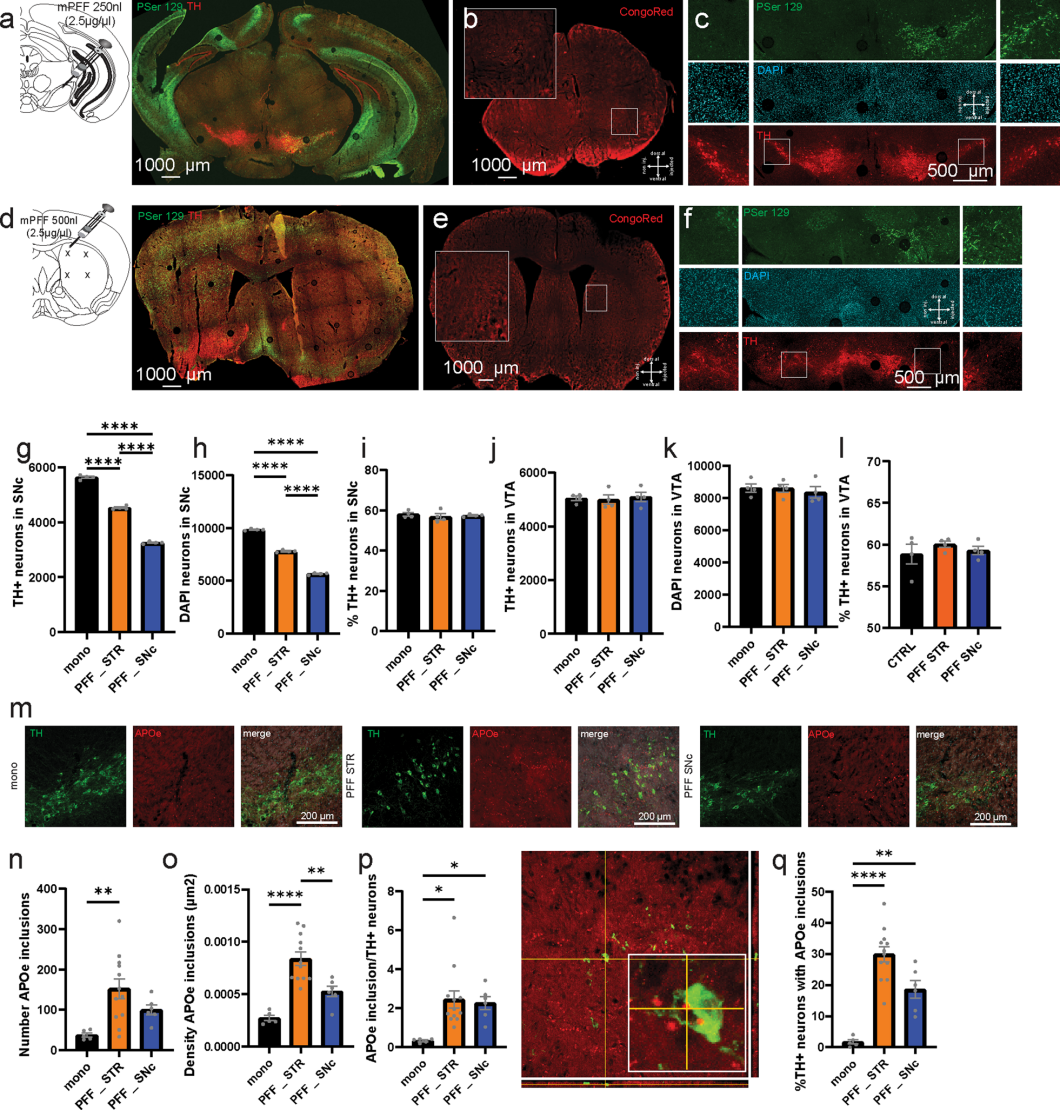
Injection of αSyn-PFFs in the SNc or striatum impacts the SNc differently. **a–f** Unilateral injection of αSyn-PFFs in the SNc (**a–c**) or the striatum (**d–f**) revealed the presence of amyloid-like structures (**b, e**) in the vicinity of the injection site. The SNc injection at the soma level (**c**) or their terminals (**f**) affects TH expression differently. **g** Number of TH-positive neurons in the SNc in WT mice injected with monomeric αSyn or αSyn-PFF in the striatum (orange) or the SNc (blue). **h** Number of DAPI-positive neurons in the SNc in WT mice injected with monomeric αSyn or αSyn-PFFs in the striatum (orange) or the SNc (blue). **i** Percentage of DAPI neurons expressing TH in the SNc in WT mice injected with monomeric αSyn or αSyn-PFF in the striatum (orange) or the SNc (blue). **j** Number of TH-positive neurons in the VTA in WT mice injected with monomeric αSyn or αSyn-PFF in the striatum (orange) or the SNc (blue). **k** Number of DAPI-positive neurons in the VTA in WT mice injected with monomeric αSyn or αSyn-PFF in the striatum (orange) or the SNc (blue). **l** Percentage of DAPI neurons expressing TH in the VTA in WT mice injected with monomeric αSyn or αSyn-PFF in the striatum (orange) or the SNc (blue). **m** Confocal images showing the presence of ApoE inclusions in the vicinity of TH-positive neurons located in the SNc, following injection of monomeric αSyn or αSyn-PFF in the striatum or SNc. **n** Total number of ApoE inclusions in the SNc of mice injected with monomeric αSyn or αSyn-PFF in the striatum (orange) or the SNc (blue). **o** Density of ApoE inclusions in the SNc of mice injected with monomeric αSyn or αSyn-PFF in the striatum (orange) or the SNc (blue). **p** Number of ApoE inclusions over the number of TH-positive neurons in the SNc of mice injected with monomeric αSyn or αSyn-PFF in the striatum (orange) or the SNc (blue). **q** Confocal images of ApoE inclusions and TH-positive neurons, with orthogonal projection showing that the inclusion is located within the neuron. Quantification of TH-positive neurons located in the SNc that contain at least one inclusion within the soma. Data are expressed as mean ± SEM. All individual points represent individual animals. *P < 0.05, **P < 0.01, ****P < 0.0001. Statistical data represent post hoc analyses compared to the monomeric αSyn group following one-way ANOVA. Histology was performed one month following injection

Viral overexpression of αSyn in the SNc suggests a role for dopamine neurons in the development of RBD-like signs in mice (Fig. 6). In PD, dopamine degeneration occurs first in terminals located in the striatum [47, 48], with the development of Lewy neurites predating the development of Lewy bodies [21, 49]. To assess whether αSyn-PFF induces dopaminergic degeneration and whether αSyn seeding in the striatum or the SNc can impact different PD-related phenotypes, focusing on sleep, we injected WT mice with αSyn-PFF in the SNc or the striatum. As a control, a group of mice was injected with monomeric αSyn. We first assessed the anatomical effects of αSyn-PFF following unilateral injection into the SNc or striatum (*n* = 4 mice in SNc, Fig. 7a–c; *n* = 4 mice in striatum, Fig. 7d–f) and we observed differential propagation of pSer129 and amyloid-likes aggregates (Fig. 7b, e). One month following injection in the SNc, we found a significant reduction of TH-expressing neurons in the SNc (Fig. 7g–i), but not in the VTA (Fig. 7j–l). Interestingly, correlating the number of TH-positive neurons to the number of DAPI-positive neurons revealed no changes, suggesting a loss of dopamine neurons rather than a mere decreased expression of dopamine neuron markers (Fig. 7i, l). Likewise, unilateral injection of αSyn-PFF in the striatum caused a reduction of TH-positive neurons in the SNc, albeit smaller than that in the SNc-injected animals (Fig. 7g–l). Altogether, these data confirmed that injection of αSyn-PFF at the somatic or terminal levels induces retrograde and anterograde spreading of pathological αSyn as well as neuronal degeneration [50].

Recently, increased expression of apolipoproteins, particularly ApoE, in dopaminergic neurons has been suggested to participate in αSyn spreading or clearance [51]. While ApoE is synthesized mainly by astrocytes, microglia, and immature neurons, neuronal ApoE levels increase in injured or stressed neurons and therefore are postulated to play a role in the growth and repair of cells in the CNS [52]. Co-staining for TH and ApoE showed that injection of αSyn-PFF in the striatum increases ApoE inclusions within the SNc (Fig. 7m–p), while the expression of ApoE inside TH^+^ neurons seems to increase regardless of whether αSyn-PFF was injected in the SNc or the striatum (Fig. 7q). This supports the idea that retrograde transport of αSyn-PFFs (i.e. injection in the striatum) induces sufficient stress onto nigrostriatal neurons.

Because the αSyn-PFF-induced degeneration of dopaminergic neurons seems to be time-dependent [50], we examined locomotor behavior of mice every two weeks after receiving bilateral injections of αSyn-PFF or monomeric αSyn in the SNc (*n* = 16 vs. 8 mice) or the striatum (*n* = 15 vs. 8 mice). In the constant speed (40 RPM) rotarod, injection of αSyn-PFF in the striatum induced a rapid reduction in the time to fall (~ 4th week post-injection) while this impairment was delayed in the mice with αSyn-PFF injection in the SNc (~ 6 weeks, Fig. S9a–d). In the docking (20 RPM) rotarod test, the time to fall decreased very quickly in both groups (~ 4th week, Fig. S9e–h). We next evaluated the animals in the cognitive flexibility task. In the FR1 task, the number of correct responses remained unchanged (Fig. S9i–l), while the number of incorrect responses increased (Fig. S9m–p). Pellet collection decreased in the animals from 4 weeks after αSyn-PFF injection in the SNc and from 6 weeks after αSyn-PFF injection in the striatum (Fig. S9q–t). Altogether, these results suggest that targeting either the terminals (i.e. αSyn-PFF in the striatum) or the soma (i.e. αSyn-PFF in the SNc) of dopamine neurons causes motor and cognitive impairments, with a temporal discrepancy.

### Targeting the soma of dopaminergic neurons with αSyn-PFF impacts RBD-like events along with modulation of dopamine release in the dorsomedial striatum

**Fig. 8:**
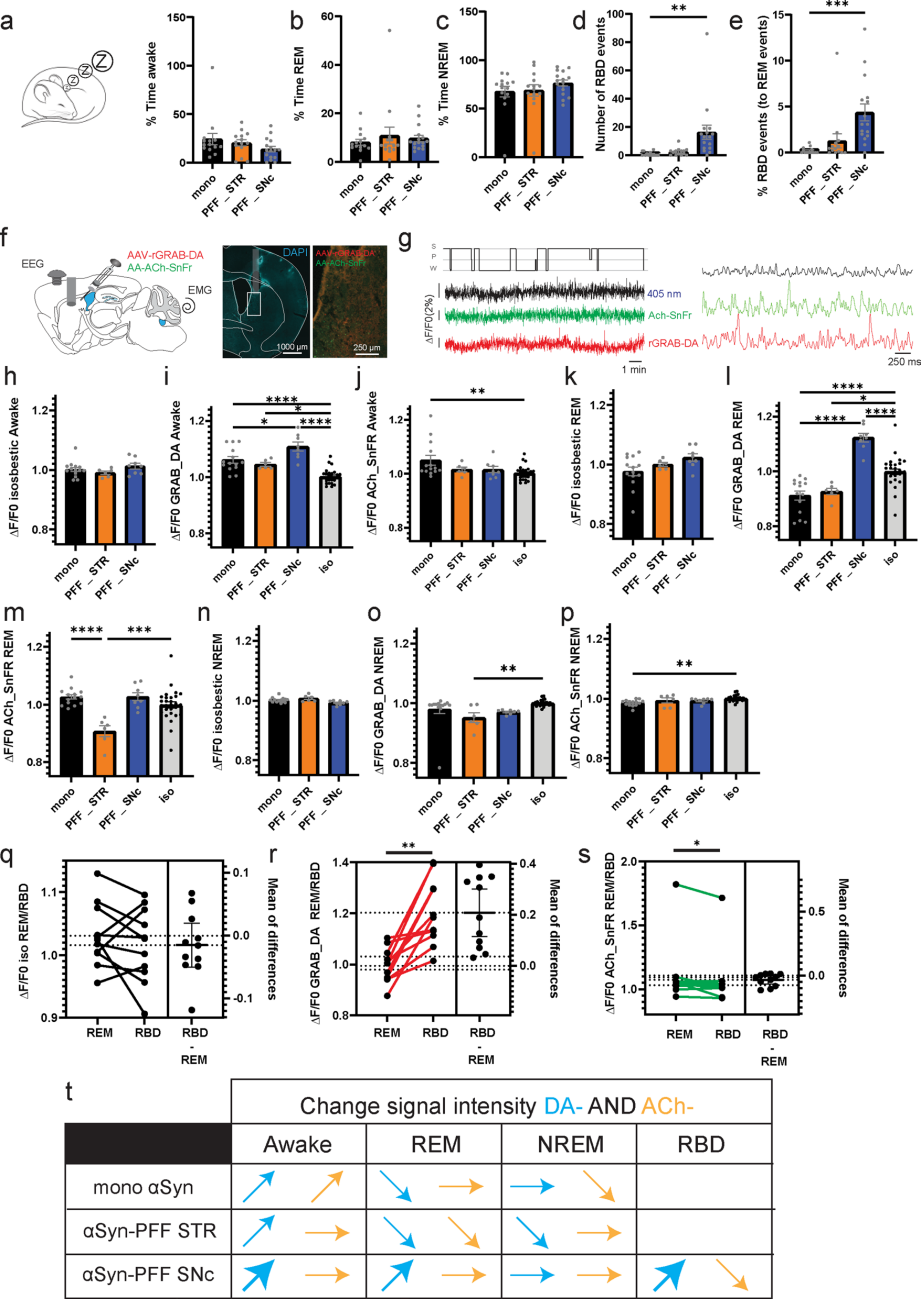
REM sleep behavior disorder is paired with alpha-synuclein aggregates in the SNc. **a–e** Percentage of time awake (**a**), in REM (**b**), or NREM (**c**), number of RBD-like events (**d**), and normalized RBD-like events relative to REM events (**e**), 8 weeks following injection of monomeric αSyn (black), or αSyn-PFF in the striatum (orange) or the SNc (blue). **f** Schematic representation of the fiber photometry experiments with recording of dopamine and acetylcholine release in the dorsomedial striatum (DMS) during sleep (left), and confocal images showing the placement of the optic fiber and the spread of the adenoviruses (right). **g** Example traces showing sleep scoring within 20-s windows, isosbestic signal, Ach-SnFr, and rGRAB-DA signal in the DMS. **h**–**p** Fiber photometry signal ΔF/F₀ of the isosbestic (**h, k, n**), rGRAB-DA (**i, l, o**), and Ach-SnFr (**j, m, p**) signals during awake (**h–j**), REM (**k–m**) and NREM (**n-p**) events in mice injected with monomeric αSyn or αSyn-PFF in the striatum (orange) or the SNc (blue). **q**–**s** Variation of the isosbestic (**q)**, rGRAB-DA (**r**), and Ach-SnFr (**s**) signals in the DMS between REM and RBD-like events in mice injected with αSyn-PFF in the SNc. **t** Summary of signal variation during the different sleep stages in monomeric αSyn- or αSyn-PFF-injected animals. Data are expressed as mean ± SEM. All individual points represent individual animals. *P < 0.05, **P < 0.01, ***P < 0.001, ****P < 0.0001. Statistical data represent post hoc analyses compared to the monomeric αSyn group following one-way ANOVA or paired *t*-tests for photometry data

We next examined whether αSyn-PFFs injected in the SNc (*n* = 16) or striatum (*n* = 14) affect sleep parameters. For comparison, mice were injected with monomeric αSyn (*n* = 6 SNc and *n* = 8 striatum). Sleep patterns were recorded using telemetry implant at 8 weeks post-injection. We found no significant difference in the patterns of awake (Fig. 8a, S10a–c), REM (Fig. 8b, S10d–f), or NREM (Fig. 8c, S10g–i) events following injection of αSyn-PFFs in the striatum or the SNc. However, quantification of the number of REM events with atonia suggested an increase in RBD-like events exclusively in mice injected with αSyn-PFFs in the SNc (Fig. 8d, e), despite the absence of general effects on the EMG signal during REM sleep (Fig. S10j). To test whether the effects observed were due to alterations of dopamine or acetylcholine release in the striatum, we injected animals with a mix of AAVs, the first being green-shifted to measure acetylcholine release (AAV-Ach-SnFr) and the second being red-shifted to measure dopamine release (AAV-rGRAB-DA), and EEG and EMG recordings were made (Fig. 8f, g). Based on a previous report on the role of dopamine release in the dorsomedial striatum during sleep, an optic fiber was then placed above the injection site [53, 54]. We next recorded sleep and photometry, including a control isosbestic signal. Sleep schedule was then scored on 20-s windows, and each photometry signal was averaged for each sleep-scored window (Fig. 8h–p). Isosbestic signals in control, striatum- or SNc-injected animals were not different during awake (Fig. 8h), REM (Fig. 8k), or NREM (Fig. 8n) events. However, pooling together their average to compare to other signal variations, awake events showed a significant increase in dopamine release in all three groups compared to the isosbestic signal (Fig. 8i, t), with the highest increase found in the SNc αSyn-PFF-injected mice. In the same awake events, we found that acetylcholine release in the striatum increased in control (i.e., monomeric αSyn-injected mice), but not in SNc- or STR-αSyn-PFF-injected mice, compared to isosbestic signal (Fig. 8j, t).

During REM events, we found that dopamine release was reduced in monomeric αSyn animals as well as in animals injected with αSyn-PFF in the striatum (Fig. 8l, t). However, the striatal release of dopamine remained high in animals injected with αSyn-PFF in the SNc (Fig. 8l, t). Acetylcholine release was unchanged in the striatum during REM events in mice injected with αSyn-PFF in the SNc (Fig. 8m, t), whereas injection of αSyn-PFF in the striatum reduced the cholinergic signal (Fig. 8m, t).

During NREM events, the dopamine release in the striatum did not change significantly in animals injected with αSyn-PFFs in the SNc compared to isosbestic variation or monomeric αSyn-injected mice, while dopamine release decreased in animals injected with αSyn-PFFs in the striatum compared to isosbestic variation (Fig. 8o, t). Finally, we found that acetylcholine release slightly decreased in monomeric αSyn-injected, but not in αSyn-PFF-injected animals, compared to isosbestic variation during NREM events (Fig. 8p, t).

As animals injected with αSyn-PFFs in the SNc demonstrated an increase of dopamine release during REM events compared to isosbestic signal as well as monomeric αSyn mice signals, together with an increase in RBD-like signs, we hypothesized that the effect could be explained by movement during REM events. To assess this, we compared photometry signals during REM with atonia and RBD-like events in SNc-injected animals (Fig. 8q–t). Not surprisingly, we found no significant difference in isosbestic signal during REM and RBD-like events (Fig. 8q). However, in both dopamine and acetylcholine signals, we found a significant difference between REM and RBD-like events, where the dopamine signal increased markedly during RBD-like events (Fig. 8r, t), while the acetylcholine signal decreased (Fig. 8s, t). These data suggest that the increase in dopamine release during REM events occurs mainly during RBD-like events.

## Discussion

In this study, we demonstrated the spread of pathological αSyn from the gut to the brain that temporally correlated with the development of non-motor and motor symptoms, particularly RBD-like signs.

### The DMV is the entry gate of the gut-brain pathway

Based on post-mortem analyses, Braak and colleagues suggested that PD might originate in the periphery and propagate to the brain via the vagus nerve [7]. Our data support the notion that pathological αSyn induced by stomach/duodenum injections of αSyn-PFF propagates from the stomach to the brain [10]. There is some debate on the circuits involved in the αSyn transmission from the enteric nervous system to the brain [10, 30, 55–57]. In recent reports using αSyn-PFF injection in the duodenum, a vagotomy was sufficient to prevent most of the phenotypes linked with αSyn aggregation [10, 58]. We observed an increase in pS129-αSyn intensity in the DMV one month after injection, with results similar to previous observations confirming a preferential propagation throughout the vagus nerve [10, 58]. In addition, at that stage we observed a significant reduction in the number of cholinergic neurons expressing ChAT (ChAT^+^ staining) within the DMV, suggesting an early dysfunction of cholinergic transmission in this structure.

### Structural associations to sleep dysfunctions, particularly RBD

Our findings highlight the role of αSyn in the gut-brain axis, demonstrating that the spread of aggregated αSyn from the gut to the brain is closely associated with the impairment of brain regions involved in the non-motor phenotypes observed in PD [7]. This raises the question of a possible degenerative pathway between the gut and the SNc involving several brain structures and their respective functions [21]. However, in our whole brain mapping, not all structures were found to accumulate αSyn following a caudal-to-rostral pattern. In particular, it is interesting to note that the gigantocellular and the medullary nuclei, both located near the DMV and connected to it [59], exhibited increased pS129-αSyn staining in the very late stages of the model, when motor manifestations were present. This discrepancy could relate to the selective neuronal vulnerability in PD [60]. Furthermore, our study takes temporal dynamics of αSyn propagation into account, revealing the concurrent presence of phosphorylated Ser129-αSyn form in key brain regions associated with sleep duration, motor function, and cognitive planning.

Our data suggest that RBD-like behavior, characterized by an absence of atonia during REM sleep, robustly occurs in the gut-brain PFF model. A previous study demonstrated that injections of αSyn-PFFs or inactivation of neurons in the sublaterodorsal tegmental nucleus (i.e., the subcoeruleus nucleus in humans) can induce RBD-like behaviors in animals [61]. The authors also found transneuronal spread of αSyn pathology to several other brain regions, and the mice developed PD-like locomotor dysfunction, depression-like disorder, olfactory dysfunction, and gastrointestinal dysmotility, suggesting a potential path for phenoconversion of RBD to motor signs of parkinsonism. In their study, the authors used toxin- or αSyn-PFF-induced degeneration of the circuit linking the brainstem and the gigantocellular nuclei and found an increase in shorter REM events [61], together with an increase in RBD-like events. As the sleep recordings occurred between 1 and 3 months following injections, and these structures are interconnected with dopaminergic neurons of the SNc [62], alterations of dopaminergic function might have already occurred [63]. In another study, using A53T *SNCA* transgenic mice, RBD-like behaviors were observed at 5 months of age [64]. These RBD-like behaviors correlated with pS129-αSyn expression in the sublaterodorsal tegmental nucleus along with nuclei in the ventromedial medullary reticular formation and the pedunculopontine nuclei [65]. This is in line with recent case studies in idiopathic RBD patients, where αSyn pathology was observed in similar structures including the LDT, but also SNc, locus coeruleus and gigantocellular nucleus [66].

In the present study, we did not specifically target the sublaterodorsal tegmental nucleus. However, our work highlights the role of dopaminergic structures in RBD-like behaviors in mice. Interestingly, targeting nigral dopaminergic neurons or their striatal terminals had no comparable influence on sleep structure when contrasted with stomach injections while recapitulating RBD-like events. This may suggest that the SNc per se or structures interconnected with this region, including the sublaterodorsal tegmental nucleus, are implicated in RBD-like behaviors. This is in line with previous observations demonstrating the reduction of the dopamine transporter in the brains of patients with RBD [67, 68] and the increase in RBD in mouse transgenic models targeting SNc neurons [64]. Moreover, our data using fiber photometry suggest that while dopamine release in the dorsomedial striatum is involved in RBD generation, acetylcholine release might be crucial for wakefulness and the switch between REM and NREM sleep, observations linking the finely tuned balance between dopamine and acetylcholine signaling in the dorsomedial striatum to sleep stage regulation, including RBD [54, 69, 70]. Crucially, while the majority of acetylcholine in the striatum originates from interneurons, a subset is released from cholinergic neurons of the brainstem including the pedunculopontine and the LDT [62, 71, 72], two structures involved in slow-wave activity and sleep regulation [73]. Altogether, these data suggest that RBD-like phenotypes in mice are initiated with a loss of function or degeneration of nigrostriatal dopaminergic neurons [74]. It is noteworthy that while non-motor manifestations can occur years before motor manifestation and diagnosis, most studies revealed a partial loss of the dopamine transporter in the striatum independently of motor alteration.

### αSyn-PFF model compared to clinical observation in the PD

Lewy pathology, progressive neurodegeneration of the nigrostriatal pathway, motor and non-motor symptoms, are key hallmarks of PD [21]. At motor manifestation, an estimated ~ 50% loss of nigrostriatal dopaminergic terminals is observed, while ~ 80% loss is commonly found post-mortem when diagnosis is confirmed [21]. Our model of αSyn PFF injection in the stomach demonstrated several key features of idiopathic PD. The peak of phosphorylated αSyn observed at 3 months post-injection, co-occurred with nigrostriatal neurons displaying a reduction of dopaminergic phenotype (i.e. TH immunoreactivity) and was then followed by neurodegeneration observed around 6 months post-injection. This process has also been observed in post-mortem tissues of PD patients [75] and linked to the downregulation of several transcription factors [76]. Along with the reduction in dopaminergic phenotypes by 30–50% we observed the development of non-motor manifestations, while motor manifestations were mostly visible in conjunction with the reduction of nigrostriatal neurons.

In our study, we tried to recapitulate several common non-motor and motor manifestations observed in PD patients, using rodent-adjusted behavioral tests. This allowed us to define that most PD features observed in our model can be aligned with either nucleus-specific aggregation of αSyn, circuit phenotypes, or neurodegeneration. Another interesting point to consider is the preliminary sex differences that we observed in our results. We found that males display faster and more pronounced motor alterations than females, with no significant differences found in non-motor phenotypes. This is in line with several observations demonstrating significant differences in PD manifestation between males and females [77]. However, it is important to note the limitations of our approach where sex analyses reduce the group size and affect the observations. Specific follow-up studies are requested to study sex difference in the model.

### Limitations of the study

The αSyn-PFF model with injection in the muscularis layers of the stomach and the duodenum and propagation to the brain appears to be a clinically relevant model in both rats [79] and mice [80–82]. However, it should be noted that under some conditions the model fails to show the progression of the disease [78] or the propagation beyond the pons [58]. One limitation of our approach is that the laparotomy during PFF injection induces local inflammation that prevents investigation within 2 weeks after PFF injection. Additionally, we cannot exclude the possibility that the injection induces local bleeding, which allows some propagation through the bloodstream. A second limitation is that, while pS129-αSyn is considered a marker for αSyn pathology, it is also present in a small, but significant, baseline amount in the CNS [15] and participates in synaptic function [6]. This makes it difficult to estimate smaller pS129-αSyn increases at early timepoints. To reduce the impact of this limitation, all our experiments included a group of animals injected with monomeric αSyn and all mouse tissues were processed and stained at the same time using the same solutions. A third limitation is the possible role of age in αSyn spreading [11]. In their work, Challis and colleagues found that aged animals (16 months or older) display αSyn propagation and phenotype progression. This is likely due to the fact that native αSyn increases with age, thereby increasing seeding between native αSyn and PFFs. To overcome a possible age-related effect, we started behavioral testing at the same age (9–10 months) and time. To achieve this, the age at injection differed across experimental groups (Fig. 1c). In addition, we divided our control groups into those injected with monomeric αSyn 2 weeks or 7 months before testing to avoid effects related to age at injection and surgery. In future work, it would be of interest to use other animal models that either present abnormal αSyn expression or have a lifespan exceeding ~ 2 years. A fourth limitation concerns the technical challenges associated with large-scale brain section imaging, where the precise alignment across different brain structures, subjects and groups is often challenging. Despite employing automated computational alignment and proportional adjustments (scaling, rotation), perfect overlap between sections is hard to achieve. This variability arises from inherent inter-animal brain size differences, sectioning artifacts (e.g., curled or lost tissue), and immunostaining variability. Since misalignments are evident at the borders of certain regions (e.g., superficial cortical layers), our analyses did not draw conclusions from small, isolated ROIs or edge-related group differences. Instead, we focused on robust changes across large, neighboring ROIs and on clearly defined nuclei, as also supported by manual delineation of anatomical landmarks. Importantly, we did not apply local warping or modifications that could distort image quality; rather, identical alignment procedures were applied across all groups, ensuring similar variability within and between groups. Thus, although misalignment represents an intrinsic limitation of large-scale imaging in large cohorts, our methodological choices mitigate its impact, allowing for group-level comparisons.

Finally, since stomach injections of αSyn-PFFs resulted in propagation of pS129-αSyn and histopathology in SNc along with non-motor symptoms, such as RBD-like events, we wanted to examine effects of local administration of αSyn in SNc on RBD-like events. We used two approaches, AAV-SNCA [44, 83] and αSyn-PFFs. There are limitations of both approaches. First, we induced human *SNCA* expression in the SNc using local administration of AAV. Because human αSyn fibrils  do not normally seed or spread in wild-type mice [84], this approach restricts pathology to the injection site. However, since we used a non-conditional AAV vector, it is likely that it drives expression not only in SNc, but also in neighboring regions (e.g., SNr, VTA, red nucleus), which may have contributed to detected phenotypes. Second, we locally injected αSyn-PFFs into the SNc or striatum. While both approaches induced pS129-αSyn and dopaminergic loss in the SNc, the spread patterns differed between injection sites, raising the possibility that histopathology in interconnected structures contributed to non-motor symptoms. Nevertheless, although our viral and PFF injection approaches of αSyn may not exclusively target dopaminergic neurons, the convergence of our findings with human imaging data argue that SNc dopaminergic pathology plays a key role in RBD.

## Conclusion

In conclusion, our findings offer an extended perspective on the pathophysiology and progression of experimental αSyn-mediated Parkinsonism, emphasizing the interconnections among gastrointestinal, neurological, and behavioral aspects. Our data emphasize a crucial role of the dopamine and acetylcholine interplay in modulating sleep stages and indicate that early, moderate degeneration of dopaminergic neurons may participate in RBD.

## Supplementary Information

Additional file 1. **Fig. S1** Confocal images of transverse sections of the stomach 1 month following injections at 2 different levels (α and β) showing staining of pS129-αSyn, ChAT, TH, and DAPI. **Fig. S2** Semi-automated whole brain mapping of pSer-129-αSyn staining. **Fig. S3** Behavioral results of control groups. **Fig. S4** Detailed effects of αSyn-PFF injections on sleep parameters. **Fig. S5** Detailed effects of αSyn-PFF injections on non-motor behavioral tests. **Fig. S6** Detailed effects of αSyn-PFF injections on motor behavioral tests. **Fig. S7** Detailed effects of αSyn-PFF injections on cognitive behavioral tests. **Fig. S8** Detailed sex effects of αSyn-PFF injections in multiple brain regions and in behavioral tests. **Fig. S9** Detailed behavioral effects of αSyn-PFF injections in striatum or SNc. **Fig. S10** Detailed effects of αSyn-PFF injections in striatum or SNc on sleep parameters.

Additional file 2. **Table S1**. Statistical analysis of p-Ser129-αSyn in different brain regions at different time points, as shown in Fig. 2

Additional file 3. **Table S2**. Excel file representing the number of animals in each group for all animal experiments.

## Abbreviations

αSyn: Alpha-synuclein
ApoE: Apolipoprotein E
CNS: Central nervous system
ChAT: Choline acetyltransferase
DMV: Dorsal motor nuclei of the vagus nerve
EEG: Electroencephalogram
FR1: Fixed-ratio 1
FR3: Fixed-ratio 3
Gi: Gigantocellular nuclei
Gpe: Globus pallidus
LDT: Laterodorsal tegmental nucleus
EMG: Neck-muscles electromyogram
PD: Parkinson’s disease
PFF: Preformed fibrils
REM: Rapid eye movement
ROIs: Region of interests
RBD: REM sleep behavior disorder
RPM: Rotations per minute
SNc: Substantia nigra pars compacta
NTS: Tractus solitarus
TH: Tyrosine hydroxylase
WT: Wild-type
pS129-αSyn: αSyn phosphorylated at serine 129
