# Supplementary material for: Gut-initiated alpha synuclein fibrils drive parkinsonism phenotypes: temporal mapping of REM sleep behavior disorder-like and other non-motor symptoms
Source: Transl Neurodegener. 2026 Mar 10;15:10. doi: 10.1186/s40035-026-00536-6 (PMC12973632; doi:10.1186/s40035-026-00536-6)
Supplement: Supplementary file 1 — Additional file 1. Fig. S1 Confocal images of transverse sections of the stomach 1 month following injections at 2 different levels (α and β) showing staining of pS129-αSyn, ChAT, TH, and DAPI. Fig. S2 Semi-automated whole brain mapping of pSer-129-αSyn staining. Fig. S3 Behavioral results of control groups. Fig. S4 Detailed effects of αSyn-PFF injections on sleep parameters. Fig. S5 Detailed effects of αSyn-PFF injections on non-motor behavioral tests. Fig. S6 Detailed effects of αSyn-PFF injections on motor behavioral tests. Fig. S7 Detailed effects of αSyn-PFF injections on cognitive behavioral tests. Fig. S8 Detailed sex effects of αSyn-PFF injections in multiple brain regions and in behavioral tests. Fig. S9 Detailed behavioral effects of αSyn-PFF injections in striatum or SNc. Fig. S10 Detailed effects of αSyn-PFF injections in striatum or SNc on sleep parameters. [file 40035_2026_536_MOESM1_ESM.pdf]

Gut-Initiated alpha synuclein fibrils drive parkinsonism phenotypes: temporal mapping of REM sleep behavior disorder-like and other non-motor symptoms

Daniel Dautan.[1,2*], Wojciech Paslawski.[1,2], Sergio G. Montejo.[1], Daniel C. Doyon.[1], Valentina I. Brioschi. [1], Roberta Marongiu[2,3], Michael G. Kaplitt.[2,3], Rong Chen.[2,4,5], Valina L. Dawson.[2,4,5,6,7], Xiaoqun Zhang.[1], Ted M. Dawson.[2,4,5,7,8], Per Svenningsson.[1,2*]

[1]Department of Clinical Neuroscience, Karolinska Institute, Stockholm, Sweden

[2]Aligning Science Across Parkinson's (ASAP) Collaborative Research Network, Chevy Chase, MD 20815, USA

[3]Department of Neurological Surgery, Weill Cornell Medicine, New York, NY, USA

[4]Neuroregeneration and Stem Cell Programs, Institute for Cell Engineering, The Johns Hopkins University School of Medicine, Baltimore, Maryland, United States of America

[5]Department of Neurology, The Johns Hopkins University School of Medicine, Baltimore, Maryland, United States of America

[6]Department of Physiology, Johns Hopkins University School of Medicine, Baltimore, MD 21205, USA

[7]Solomon H. Snyder Department of Neuroscience, The Johns Hopkins University School of Medicine, Baltimore, Maryland, United States of America

[8]Department of Pharmacology and Molecular Sciences, The Johns Hopkins University School of Medicine, Baltimore, Maryland, United States of America

* Corresponding authors: Daniel Dautan daniel.dautan@ki.se; Per Svenningsson per.svenningsson@ki.se

Figure S1

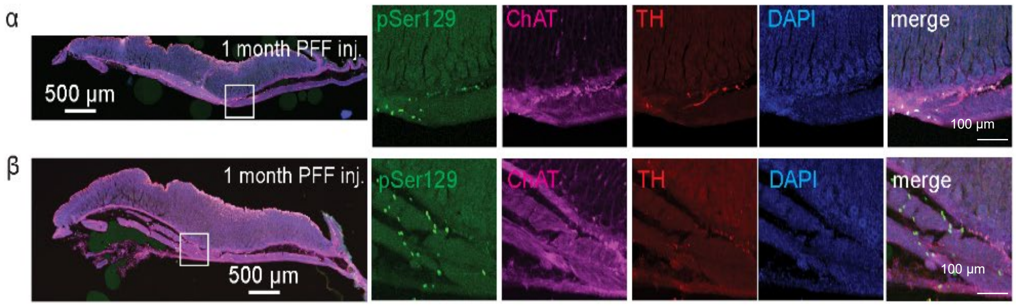

Fig. S1. Confocal images of transverse sections of the stomach 1 month following injections at 2 different levels (α and β) showing staining of pS129-αSyn, ChAT, TH, and DAPI.

Figure S2

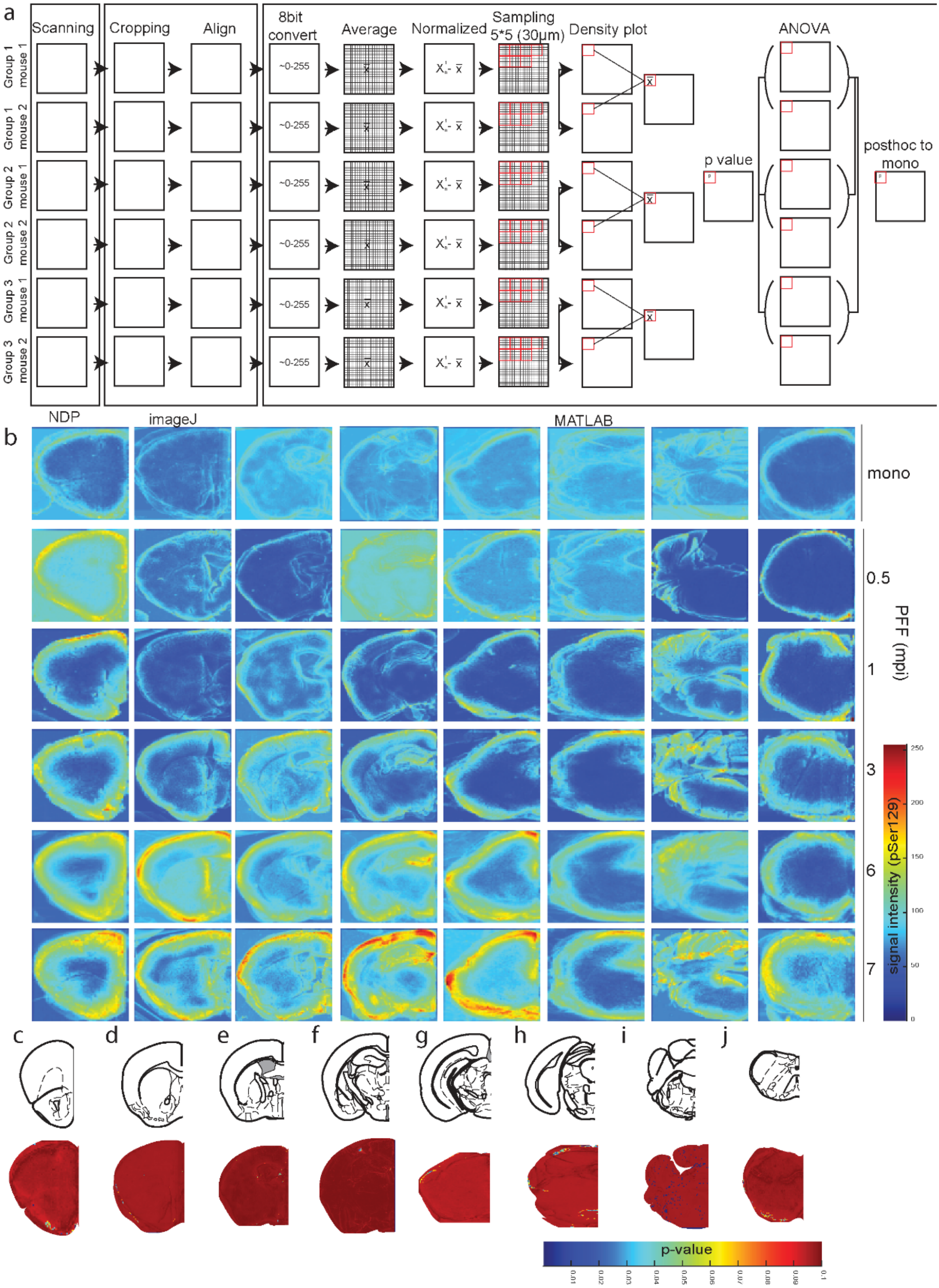
a
Scanning
Cropping
Align
8bit convert
Average
Normalized
Sampling 5*5 (30μm)
Density plot
ANOVA
p value
posthoc to mono
Group 1 mouse 1
Group 1 mouse 2
Group 2 mouse 1
Group 2 mouse 2
Group 3 mouse 1
Group 3 mouse 2
~0-255
NDP
imageJ
MATLAB
b
mono
0.5
1
3
6
7
PFF (mpi)
signal intensity (pSer129)
c
d
e
f
g
h
i
j
p-value

Fig. S2. Semi automated whole brain mapping of pSer-129-αSyn staining. a Graphical illustration of the analyses of single and group images obtained for pS129-αSyn DAB staining. **b** Representative images of the average density plot at different antero-posterior levels for mice injected with monomeric αSyn or αSyn-PFFs in the stomach. **c-j** ANOVA comparison of the ROI analyses between monomeric αSyn-injected animals at 2 weeks and 7 months.

Figure S3

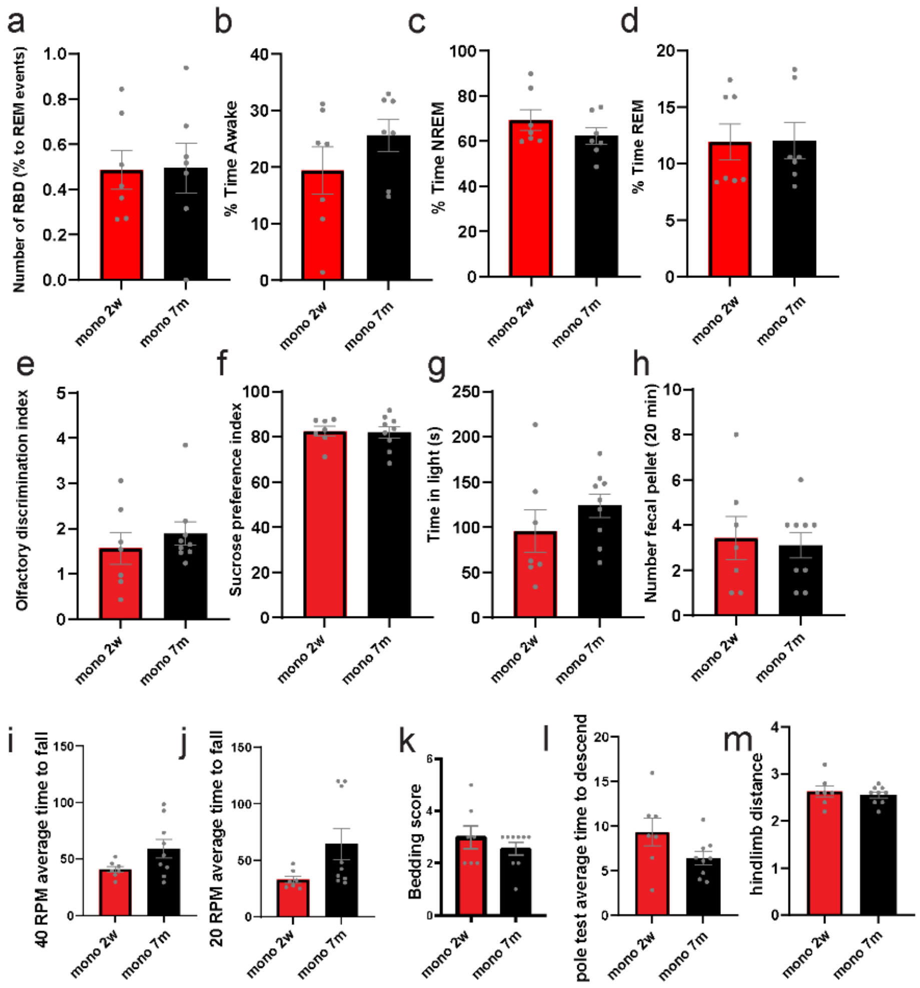

Fig. S3. Behavioral results of control groups.

a-d Percentage of RBD-like events over the number of REM events (**a**), percentage of time awake (**b**), percentage of NREM (**c**), and percentage in REM (**d**) in mice injected with monomeric αSyn for 2 weeks (red) or 7 months (black). **e** Olfactory discrimination index in mice injected with monomeric αSyn for 2 weeks (red)or 7 months (black). **f** Sucrose preference index in mice injected with monomeric αSyn for 2 weeks (red) or 7 months (black). **g** Time spent in the light area of the dark-light box in mice injected with monomeric αSyn for 2 weeks (red) or 7 months (black). **h** Number of fecal pellets in mice injected with monomeric αSyn for 2 weeks (red) or 7 months (black). **i** Time to fall on the constant-speed 40 RPM rotarod schedule between monomeric αSyn-injected animals at 2 weeks (red) or 7 months (black). **j** Time to fall on the accelerating 20 RPM rotarod schedule between monomeric αSyn-injected animals at 2

weeks (red) or 7 months (black). **k** Bedding score between monomeric αSyn-injected animals at 2 weeks (red) or 7 months (black). **l** Time to descend the pole during the pole test between monomeric αSyn-injected animals at 2 weeks (red) or 7 months (black). **m** Average hindlimb distance during the inclined platform test between monomeric αSyn-injected animals at 2 weeks (red) or 7 months (black). Data are expressed as mean ± SEM. All individual points represent individual animals. Statistical analyses were performed using unpaired t-tests.

Figure S4

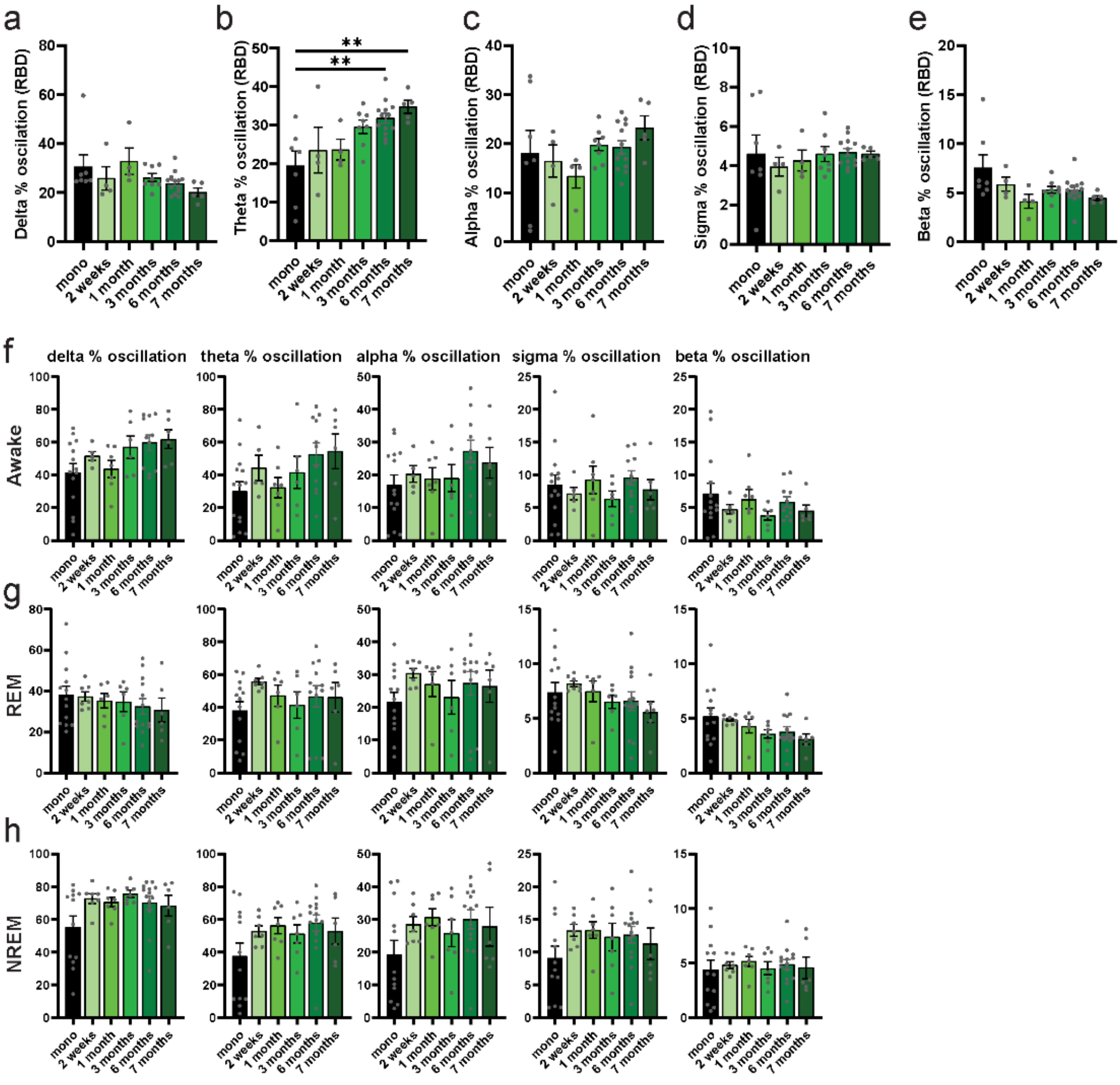
a
b
c
d
e
Delta % oscilation (RBD)
Theta % oscillation (RBD)
Alpha % oscillation (RBD)
Sigma % oscillation (RBD)
Beta % oscillation (RBD)
**
**
mono
2 weeks
1 month
3 months
6 months
7 months
f
g
h
delta % oscillation
theta % oscillation
alpha % oscillation
sigma % oscillation
beta % oscillation
Awake
REM
NREM

Fig. S4. Detailed effects of αSyn-PFF injections on sleep parameters. a Delta oscillation of the cortical periodogram, expressed as a percentage of all oscillations during RBD-like events. **b** Theta oscillation of the cortical periodogram, expressed as a percentage of all oscillations during RBD events. **c** Alpha oscillation of the cortical periodogram, expressed as a percentage of all oscillations during RBD events. **d** Sigma oscillation of the cortical periodogram, expressed as a percentage of all oscillations during RBD events. **e** Beta oscillation of the cortical periodogram, expressed as a percentage of all oscillations during RBD events. **f** Cortical periodogram oscillations (delta, theta, alpha, sigma, and beta) expressed as a percentage of all oscillations during awake events. **g** Cortical periodogram oscillations (delta, theta, alpha, sigma, and beta) expressed as a percentage of all oscillations during REM events. **h** Cortical periodogram oscillations (delta, theta, alpha, sigma, and beta) expressed as a percentage of all oscillations during NREM events. Data are expressed as mean ± SEM. All individual points represent individual animals. $^{**}P < 0.01$. Statistical analyses represent post hoc comparisons to the monomeric αSyn group following one-way ANOVA.

Figure S5

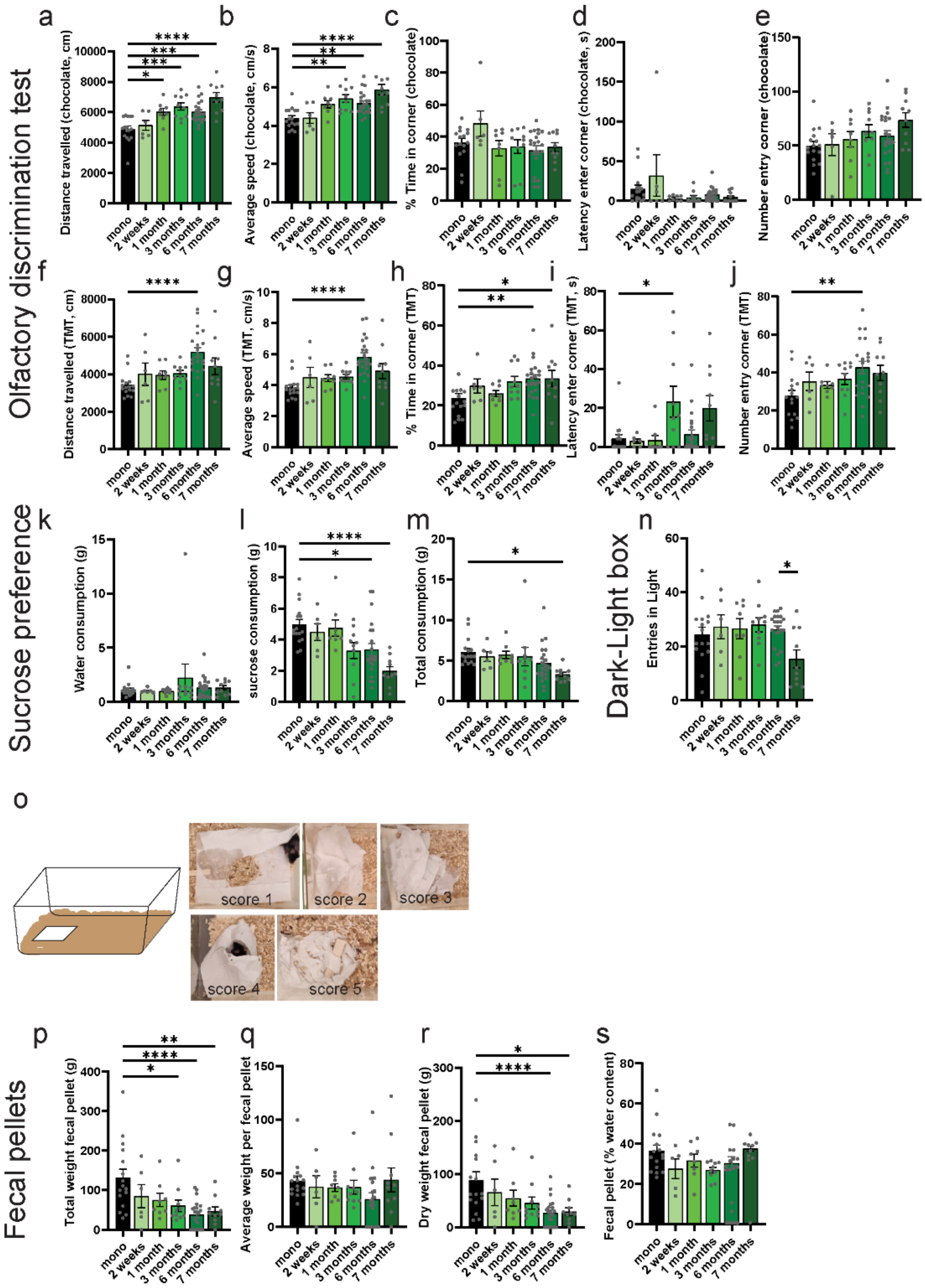

Olfactory discrimination test
Sucrose preference
Dark-Light box
Fecal pellets
a
b
c
d
e
f
g
h
i
j
k
l
m
n
o
p
q
r
s
Distance travelled (chocolate, cm)
Average speed (chocolate, cm/s)
% Time in corner (chocolate)
Latency enter corner (chocolate, s)
Number entry corner (chocolate)
Distance travelled (TMT, cm)
Average speed (TMT, cm/s)
% Time in corner (TMT)
Latency enter corner (TMT, s)
Number entry corner (TMT)
Water consumption (g)
sucrose consumption (g)
Total consumption (g)
Entries in Light
score 1
score 2
score 3
score 4
score 5
Total weight fecal pellet (g)
Average weight per fecal pellet
Dry weight fecal pellet (g)
Fecal pellet (% water content)
mono
2 weeks
1 month
3 months
6 months
7 months

Fig. S5. Detailed effects of αSyn-PFF injections on non-motor behavioral tests. a Distance traveled during the olfactory test paired with chocolate smell. **b** Average speed during the olfactory test paired with chocolate smell. **c** Percentage of time spent in the corner paired with chocolate smell. **d** Latency to the first entry in the corner paired with chocolate smell. **e** Total number of entries in the corner paired with chocolate smell. **f** Distance traveled during the olfactory test paired with TMT smell. **g** Average speed during the olfactory test paired with TMT smell. **h** Percentage of time spent in the corner paired with TMT smell. **i** Latency to the first entry in the corner paired with TMT smell. **j** Total number of entries in the corner paired with TMT smell. **k** Total water consumption over the 24h test. **l** Total sucrose consumption over the 24h test. **m** Total consumption over the 24h test. **n** Total number of entries in the light zone during the dark-light test. **o** Graphical illustration of the scoring parameters for the bedding test. **p** Total weight of fecal pellets produced in 20 min. **q** Average weight per individual pellet produced in 20 min. **r** Weight of the dry content of fecal pellets produced in 20 min. **s** Percentage of water content in fecal pellets produced in 20 min. Data are expressed as mean ± SEM. All individual points represent individual animals. *$P < 0.05$, **$P < 0.01$, ***$P < 0.001$, ****$P < 0.0001$. Statistical analyses represent post hoc comparisons to the monomeric αSyn group following one-way ANOVA.

Figure S6

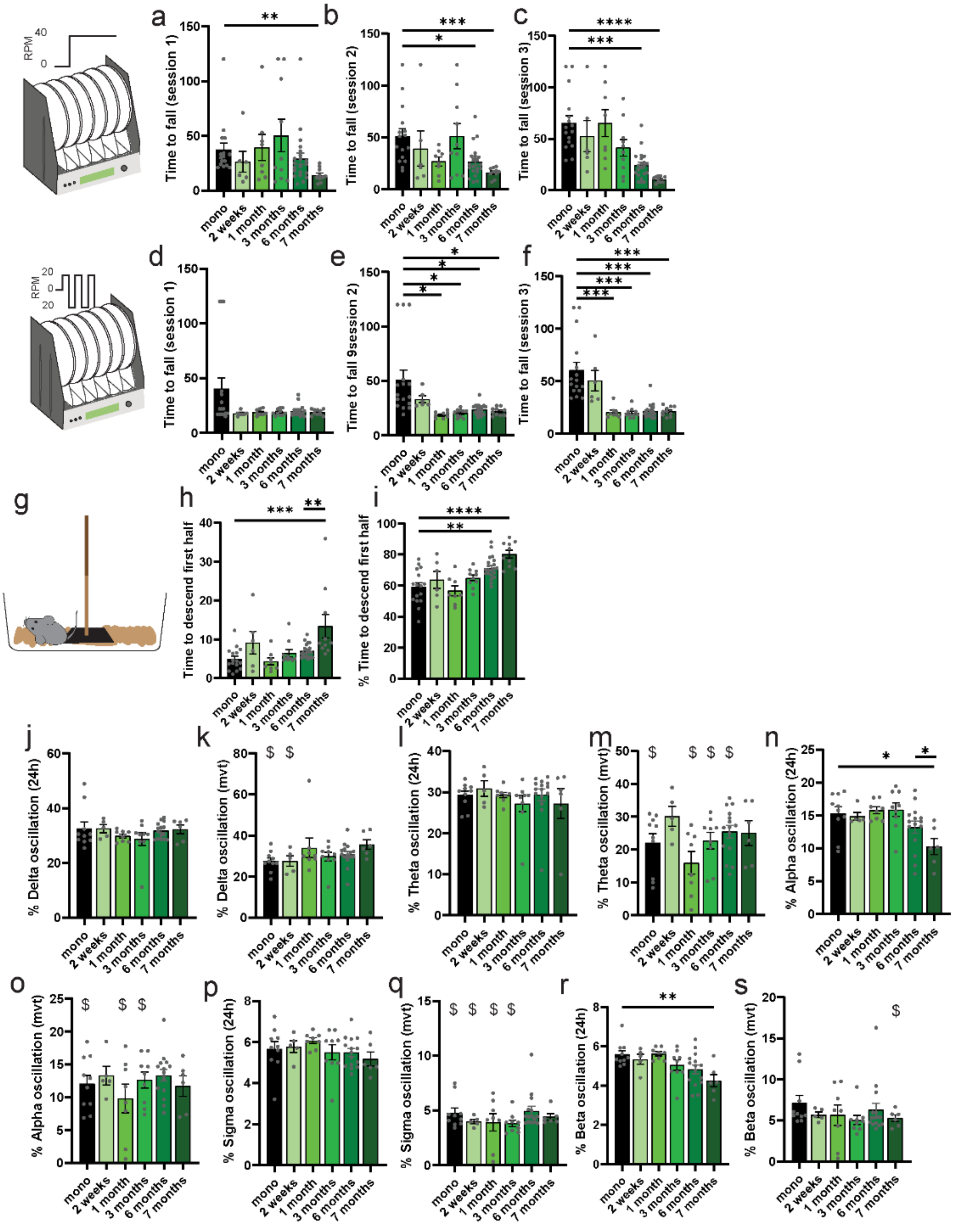

a
b
c
d
e
f
g
h
i
j
k
l
m
n
o
p
q
r
s
RPM
Time to fall (session 1)
Time to fall (session 2)
Time to fall (session 3)
Time to fall 9session 2)
Time to descend first half
% Time to descend first half
% Delta oscillation (24h)
% Delta oscillation (mvt)
% Theta oscillation (24h)
% Theta oscillation (mvt)
% Alpha oscillation (24h)
% Alpha oscillation (mvt)
% Sigma oscillation (24h)
% Sigma oscillation (mvt)
% Beta oscillation (24h)
% Beta oscillation (mvt)
mono
2 weeks
1 month
3 months
6 months
7 months

Fig. S6. Detailed effects of αSyn-PFF injections on motor behavioral tests a-f Time to fall on the constant speed 40 RPM rotarod schedule during first session (**a**), second (**b**), and third session (**c**). Time to fall on the docking speed 20 RPM rotarod schedule during first session (**d**), second (**e**), and third session (**f**). **g** Graphical illustration of the pole test. **h,i** Time to descend the first half (**h**) and percentage of time to descend the first half of the pole test compared to the entire pole test or the second of the pole during the pole test (**i**). **j,k** Delta oscillation of the cortical periodogram expressed as a percentage of all oscillations during 24-hour (**j**) or during movement (**k**). **l,m** Theta oscillation of the cortical periodogram expressed as a percentage of all oscillations during 24-hour (**l**) or during movement (**m**). **n,o** Alpha oscillation of the cortical periodogram expressed as a percentage of all oscillations during 24-hour (**n**) or during movement (**o**). **p,q** Sigma oscillation of the cortical periodogram expressed as a percentage of all oscillations during 24-hour (**p**) or during movement (**q**). **r,s** Beta oscillation of the cortical periodogram expressed as a percentage of all oscillations during 24-hour (**r**) or during movement (**s**). Data are expressed as mean ± SEM. All individual points represent individual animals. * $P < 0.05$, ** $P < 0.01$, *** $P < 0.001$, **** $P < 0.0001$. Statistical data represents post hoc analyses compared to monomeric αSyn group following one-way ANOVA. $ is used for $P < 0.05$ of the paired t-test between groups during 24h and activity events.

Figure S7

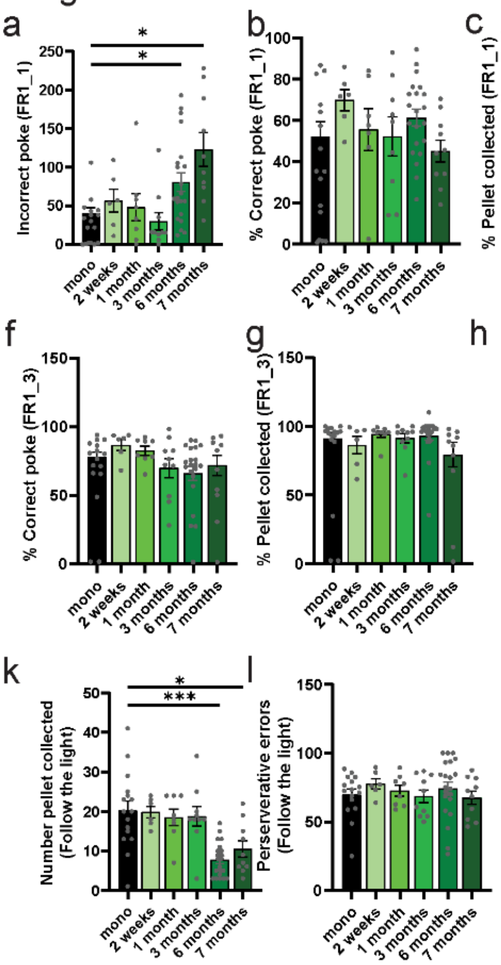

Fig. S7. Detailed effects of αSyn-PFF injections on cognitive behavioral tests. a Number of incorrect nose pokes in the first session of the fixed ratio 1. **b** Percentage of correct pokes in the first session of the fixed ratio 1. **c** Percentage of pellets collected within 10 s in the first session of the fixed ratio 1. **d** Number of correct pokes in the third session of the fixed ratio 1. **e** Number of incorrect pokes in the third session of the fixed ratio 1. **f** Percentage of correct pokes in the third session of the fixed ratio 1. **g** Percentage of pellets collected within 10 s in the third session of the fixed ratio 1. **h** Number of incorrect pokes in the first session of the fixed ratio 3. **i** Percentage of correct pokes in the first session of the fixed ratio 3. **j** Percentage of pellets collected within 10 s in the first session of the fixed ratio 3. **k** Total number of pellets collected within 10 s in the "follow the light" protocol. **l** Number of perseverative errors in the "follow the light" protocol. Data are expressed as mean ± SEM. All individual points represent

individual animals. * $P < 0.05$, *** $P < 0.0001$. Statistical data represent post hoc analyses compared to the monomeric αSyn group following one-way ANOVA.

Figure S8

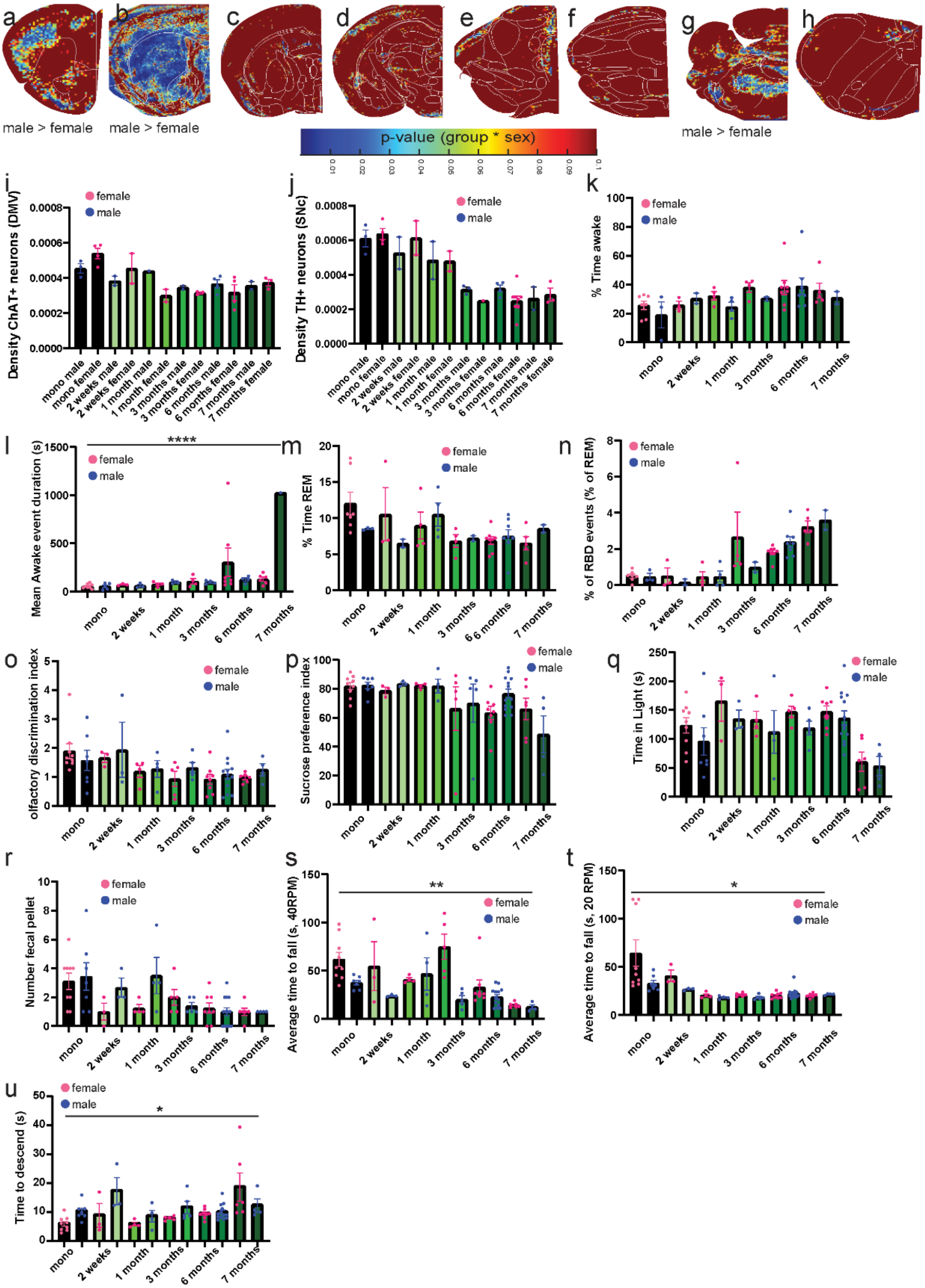

male > female
male > female
p-value (group * sex)
male > female
female
male
Density ChAT+ neurons (DMV)
Density TH+ neurons (SNc)
% Time awake
Mean Awake event duration (s)
****
% Time REM
% of RBD events (% of REM)
olfactory discrimination index
Sucrose preference index
Time in Light (s)
Number fecal pellet
Average time to fall (s, 40RPM)
**
Average time to fall (s, 20 RPM)
*
Time to descend (s)
*
mono
2 weeks
1 month
3 months
6 months
7 months

Fig. S8. Detailed sex effects of αSyn-PFF injections in multiple brain regions and in behavioral tests. a-h Two-way ANOVA comparison of the intensity of pS129-αSyn DAB staining in ROIs between male and female (sex × group effects) in (**a**) the frontal cortex, (**b**) the striatum, (**c**) the forebrain, (**d**) the thalamus, (**e**) the midbrain, (**f**) the brainstem, (**g**) the Pons, and (**h**) the medulla. **i,j** Density of ChAT-positive neurons in the DMV (**i**) or TH-positive neurons in the SNc (**j**) of male and female mice injected with monomeric αSyn or αSyn-PFF in the stomach. **k-n** Percentage of time awake (**k**), mean awake event duration (**l**), percentage of time in REM (**m**), and percentage of RBD-like events over REM events (**n**) in male and female mice injected with monomeric αSyn or αSyn-PFF in the stomach. **o-u** Olfactory discrimination index (**o**), sucrose preference index (**p**), time in the light zone during the dark-light test (**q**), number of fecal pellets (**r**), average latency to fall across all sessions of the constant-speed 40 RPM rotarod schedule (**s**), average latency to fall across all sessions of the docking-speed 20 RPM rotarod schedule (**t**), and time to descend in the pole test (**u**) in male and female mice injected with monomeric αSyn or αSyn-PFF in the stomach. Data are expressed as mean ± SEM. All individual points represent individual animals. * $P < 0.05$, ** $P < 0.01$, **** $P < 0.0001$. Statistical data represent two-way ANOVA sex × group interaction.

Figure S9

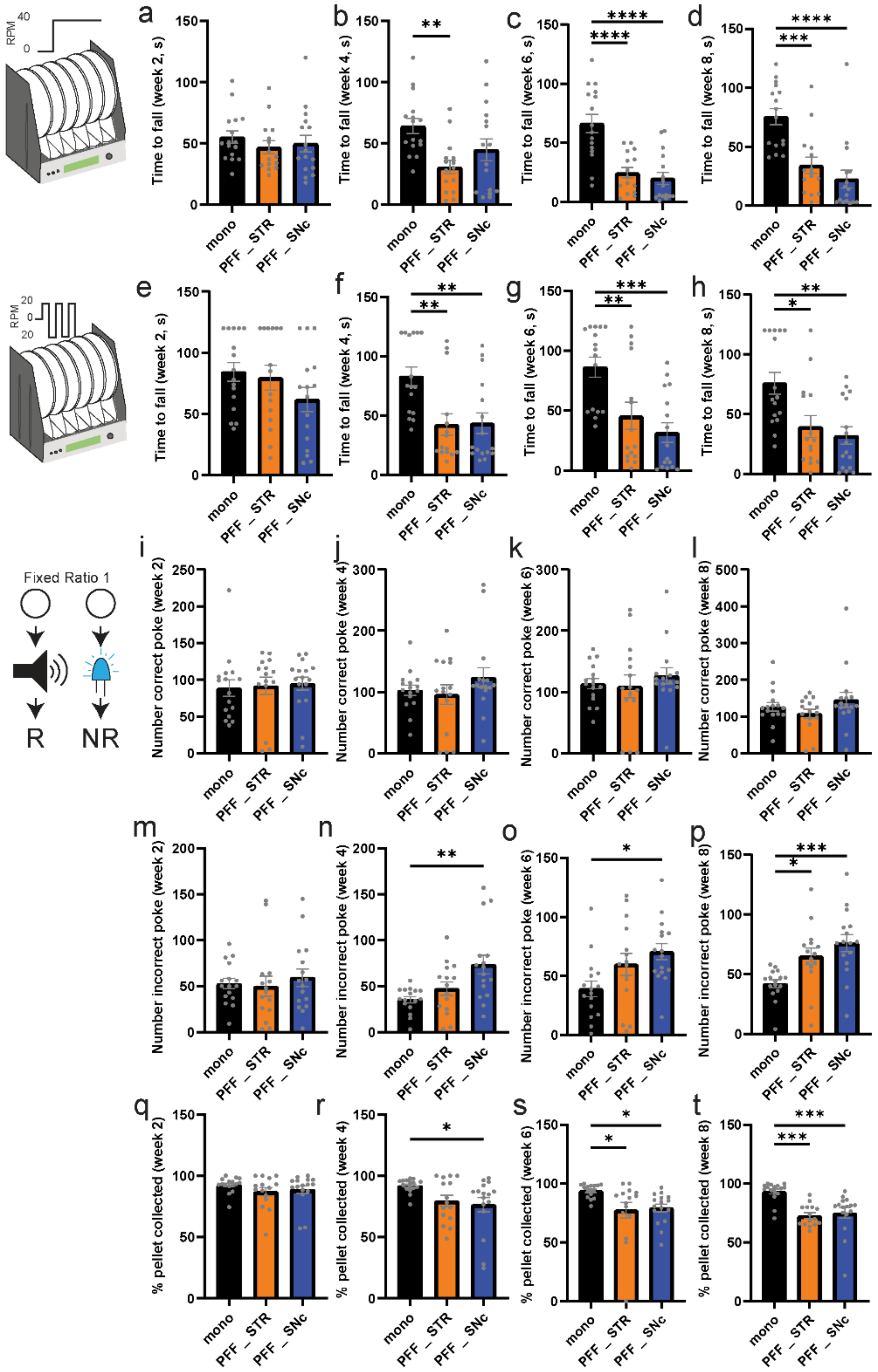

a
b
c
d
e
f
g
h
i
j
k
l
m
n
o
p
q
r
s
t
RPM
40
0
20
Fixed Ratio 1
R
NR
Time to fall (week 2, s)
Time to fall (week 4, s)
Time to fall (week 6, s)
Time to fall (week 8, s)
Number correct poke (week 2)
Number correct poke (week 4)
Number correct poke (week 6)
Number correct poke (week 8)
Number incorrect poke (week 2)
Number incorrect poke (week 4)
Number incorrect poke (week 6)
Number incorrect poke (week 8)
% pellet collected (week 2)
% pellet collected (week 4)
% pellet collected (week 6)
% pellet collected (week 8)
mono
PFF _STR
PFF _SNc

Fig. S9. Detailed behavioral effects of αSyn-PFF injections in striatum or SNc. a-d Latency to fall in the constant-speed 40 RPM rotarod schedule following 2 (**a**), 4 (**b**), 6 (**c**), or 8 (**d**) weeks post-injection of monomeric αSyn or αSyn-PFF in the striatum (orange) or the SNc (blue). **e-h** Latency to fall in the docking-speed 20 RPM rotarod schedule following 2 (**e**), 4 (**f**), 6 (**g**), or 8 (**h**) weeks post-injection of monomeric αSyn or αSyn-PFF in the striatum (orange) or the SNc (blue). **i-l** Number of correct pokes in the Fixed Ratio 1 following 2 (**i**), 4 (**j**), 6 (**k**), or 8 (**l**) weeks post-injection of monomeric αSyn or αSyn-PFF in the striatum (orange) or the SNc (blue). **m-p** Number of incorrect pokes in the Fixed Ratio 1 following 2 (**m**), 4 (**n**), 6 (**o**), or 8 (**p**) weeks post-injection of monomeric αSyn or αSyn-PFF in the striatum (orange) or the SNc (blue). **q-t** Number of pellets collected within 10 s in the Fixed Ratio 1 following 2 (**q**), 4 (**r**), 6 (**s**), or 8 (**t**) weeks post-injection of monomeric αSyn or αSyn-PFF in the striatum (orange) or the SNc (blue). Data are expressed as mean ± SEM. All individual points represent individual animals. * $P < 0.05$, ** $P < 0.01$, *** $P < 0.001$, **** $P < 0.0001$. Statistical data represent post hoc analyses compared to the monomeric αSyn group following one-way ANOVA.

Figure S10

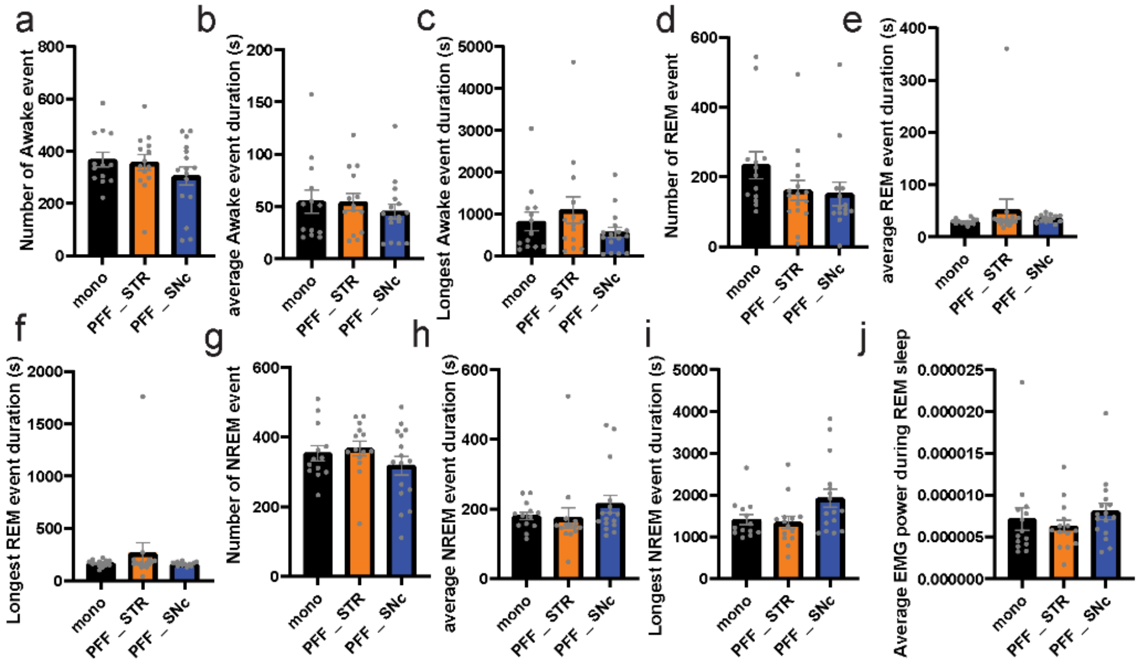

Fig. S10. Detailed effects of αSyn-PFF injections in striatum or SNc on sleep parameters

a Number of awake events during the 24h recording in mice injected with monomeric αSyn or αSyn-PFF in the striatum (orange) or the SNc (blue). **b** Average awake event duration during the 24h recording in mice injected with monomeric αSyn or αSyn-PFF in the striatum (orange) or the SNc (blue). **c** Longest awake event duration during the 24h recording in mice injected with monomeric αSyn or αSyn-PFF in the striatum (orange) or the SNc (blue). **d** Number of REM events during the 24h recording in mice injected with monomeric αSyn or αSyn-PFF in the striatum (orange) or the SNc (blue). **e** Average REM event duration during the 24h recording in mice injected with monomeric αSyn or αSyn-PFF in the striatum (orange) or the SNc (blue). **f** Longest REM event duration during the 24h recording in mice injected with monomeric αSyn or αSyn-PFF in the striatum (orange) or the SNc (blue). **g** Number of NREM events during the 24h recording in mice injected with monomeric αSyn or αSyn-PFF in the striatum (orange) or the SNc (blue). **h** Average NREM event duration during the 24h recording in mice injected with monomeric αSyn or αSyn-PFF in the striatum (orange) or the SNc (blue). **i** Longest NREM event duration during the 24h recording in mice injected with monomeric αSyn or αSyn-PFF in the striatum (orange) or the SNc (blue). **j** Average EMG signal during

REM events during the 24h recording in mice injected with monomeric αSyn or αSyn-PFF in the striatum (orange) or the SNc (blue). Data are expressed as mean ± SEM. All individual points represent individual animals. Statistical data represent post hoc analyses compared to the monomeric αSyn group following one-way ANOVA.
